# Haploinsufficiency of mechanistic target of rapamycin ameliorates *bag3* cardiomyopathy in adult zebrafish

**DOI:** 10.1242/dmm.040154

**Published:** 2019-10-01

**Authors:** Yonghe Ding, Alexey V. Dvornikov, Xiao Ma, Hong Zhang, Yong Wang, Matthew Lowerison, Rene R. Packard, Lei Wang, Jun Chen, Yuji Zhang, Tzung Hsiai, Xueying Lin, Xiaolei Xu

**Affiliations:** 1 Department of Biochemistry and Molecular Biology, Mayo Clinic, Rochester, MN 55905, USA; 2 Department of Cardiovascular Medicine, Mayo Clinic, Rochester, MN 55905, USA; 3 Center for Clinical and Translational Science, Mayo Clinic, Rochester, MN 55905, USA; 4 Mayo Graduate School of Biomedical Sciences, Mayo Clinic, Rochester, MN 55905, USA; 5 Clinical Center for Gene Diagnosis and Therapy, the Second Xiangya Hospital of Central South University, Changsha, China 410011; 6 Institute of Life Science, Beijing University of Chinese Medicine, Beijing, China 100029; 7 Department of Urology, Mayo Clinic, Rochester, MN 55905, USA; 8 School of Medicine, University of California Los Angeles, Los Angeles, CA 90073, USA; 9 Department of Epidemiology and Public Health, University of Maryland School of Medicine, Baltimore, MD 21201, USA; 10 Division of Biomedical Statistics and Informatics, Mayo Clinic, Rochester, MN 55905, USA

**Keywords:** Dilated cardiomyopathy, BCL2-associated athanogene 3, mTOR, *Danio rerio*

## Abstract

The adult zebrafish is an emerging vertebrate model for studying human cardiomyopathies; however, whether the simple zebrafish heart can model different subtypes of cardiomyopathies, such as dilated cardiomyopathy (DCM), remains elusive. Here, we generated and characterized an inherited DCM model in adult zebrafish and used this model to search for therapeutic strategies. We employed transcription activator-like effector nuclease (TALEN) genome editing technology to generate frame-shift mutants for the zebrafish ortholog of human BCL2-associated athanogene 3 (*BAG3*), an established DCM-causative gene. As in mammals, the zebrafish *bag3* homozygous mutant (*bag3^e2/e2^*) exhibited aberrant proteostasis, as indicated by impaired autophagy flux and elevated ubiquitinated protein aggregation. Through comprehensive phenotyping analysis of the mutant, we identified phenotypic traits that resembled DCM phenotypes in mammals, including cardiac chamber enlargement, reduced ejection fraction characterized by increased end-systolic volume/body weight (ESV/BW), and reduced contractile myofibril activation kinetics. Nonbiased transcriptome analysis identified the hyperactivation of the mechanistic target of rapamycin (mTOR) signaling in *bag3^e2/e2^* mutant hearts. Further genetic studies showed that *mtor^xu015/+^*, an mTOR haploinsufficiency mutant, repaired abnormal proteostasis, improved cardiac function and rescued the survival of the *bag3^e2/e2^* mutant. This study established the *bag3^e2/e2^* mutant as a DCM model in adult zebrafish and suggested *mtor* as a candidate therapeutic target gene for *BAG3* cardiomyopathy.

## Abstract (precis)

**Summary:** This study shows that adult *bag3* knockout mutant zebrafish can be used as a model for DCM, and haploinsufficiency of mTOR is cardioprotective.

## INTRODUCTION

Dilated cardiomyopathy (DCM) is a heterogeneous group of cardiac diseases characterized by an enlarged ventricular chamber, thinned ventricular walls and reduced cardiac function (Japp et al., 2016; Merlo et al., 2018). More than 100 genes have been linked to DCM. Mutations in sarcomeric genes, such as titin, account for >25% of inherited DCMs (Herman et al., 2012). Mutations in many other genes, which encode cytoskeletal, mitochondrial, desmosomal, nuclear membrane and RNA-binding proteins, have also been identified in DCM cases (McNally and Mestroni, 2017). Thus, the primary damage to the heart must be highly different among DCM subtypes with distinct etiologies. Nevertheless, the initial damage can trigger cascades of sequential pathological events that converge on certain common pathological pathways, which eventually result in DCM phenotypes. Uncovering common pathological pathways of DCM promises the development of shared therapeutic strategies for different subtypes of DCMs.

BCL2-associated athanogene 3 (*BAG3*) mutations were initially linked to DCM via two genome-wide association studies (Norton et al., 2011; Villard et al., 2011). Subsequent human genetic studies established *BAG3* as one of the most common DCM causative genes, with its variants contributing to 2.3-6.7% of DCMs (Dominguez et al., 2018; Franaszczyk et al., 2014). *BAG3* cardiomyopathy is likely of a loss-of-function nature because truncation mutations in *BAG3* are frequently found in DCM patients, and a cardiac-specific *Bag3-*knockout mouse manifests DCM phenotypes (Chami et al., 2014; Dominguez et al., 2018; Fang et al., 2017; Franaszczyk et al., 2014). Multiple functions have been assigned to the BAG3 protein: it is a co-chaperone protein that binds heat shock protein 70 (HSP70) family members and regulates protein aggregation (Meriin et al., 2018; Sturner and Behl, 2017); a BCL2-binding protein that controls apoptosis (Lee et al., 1999); and a Z-disc protein that is involved in sarcomeric protein turnover (Arimura et al., 2011; Hishiya et al., 2010). Because these functions are related to protein quality control (PQC), abnormal protein homeostasis has been postulated as the primary damage that causes DCM in patients with *BAG3* mutations (Myers et al., 2018). Although repairing defective proteostasis could be a plausible therapeutic strategy, no target genes have yet been reported for *BAG3* cardiomyopathy.

Mechanistic target of rapamycin (mTOR) is a serine/threonine protein kinase that plays a pivotal role in regulating proteostasis in cardiomyocytes by regulating cardiomyocyte growth, autophagy and survival (Saxton and Sabatini, 2017; Sciarretta et al., 2018). mTOR signaling was previously perceived as a pathway involved in physiological hypertrophy (Maillet et al., 2013). Accumulating evidence suggests that mTOR signaling can also be manipulated to benefit pathological cardiomyopathies (Sciarretta et al., 2014; Song et al., 2010). Elevated mTOR activity was detected in cardiac hypertrophy and ischemia/reperfusion-induced heart injury (Sciarretta et al., 2018). Partial mTOR inhibition through either pharmacologic or genetic inhibition exerted cardioprotective effects on several subtypes of cardiomyopathies, such as cardiac hypertrophy (Marin et al., 2011; McMullen et al., 2004), lamin A/C-deficient DCM (Ramos et al., 2012), and anemia and doxorubicin-induced cardiomyopathies (DIC) (Ding et al., 2011). Whether mTOR inhibition is effective in ameliorating the *BAG3* cardiomyopathy subtype remains untested.

Because of the unprecedented opportunities to conduct both genetic and compound screening, adult zebrafish have recently been developed as an emerging vertebrate model for human cardiomyopathy (Gut et al., 2017; Henke et al., 2017; MacRae and Peterson, 2015). Corresponding orthologs for most known human DCM genes (96%) have been identified in zebrafish (Shih et al., 2015). Conserved cardiac remodeling responses occur when fish hearts are stressed by either chronic anemia or the chemotherapy drug doxorubicin (Ding et al., 2011), and a *titin* truncation mutant in zebrafish exhibits cardiomyopathy-like phenotypes (Huttner et al., 2018). However, owing to its small body size and sponge-like heart structure, phenotyping cardiomyopathy in adult zebrafish remains a challenging task. As a consequence, the characteristic DCM phenotypes and whether different subtypes of DCM can be discerned in this simple vertebrate model remain unclear.

Here, we report the generation of a zebrafish model of *bag3* cardiomyopathy via genome editing technology. Utilizing emerging technologies, such as high-frequency echocardiography (HFE) (Wang et al., 2017), our newly developed *ex vivo* heart pump function assay (Zhang et al., 2018), and biophysical assays at the single-myofibril level (Dvornikov et al., 2014), we characterized phenotypic traits comprehensively in the *bag3* mutant. By comparison with other existing cardiomyopathy models, we proposed phenotypic traits that could be used to define DCM in an adult zebrafish. We show that the mTOR pathway is hyperactive in the *bag3* mutant, and partial mTOR inhibition exerts a cardioprotective effect on this particular subtype of inherited cardiomyopathy.

## RESULTS

### Generation of *bag3* mutations in zebrafish

In zebrafish, there is a single ortholog of the human *BAG3* gene on chromosome 13. The *bag3* gene encodes a protein that shares 55% similarity with the human BAG3 protein and up to 97% identity in functional domains, such as the WW domain (Fig. S1). The zebrafish *bag3* transcripts are enriched in striated muscles during embryogenesis and are more predominantly expressed in the cardiac muscle than in the somites in adults (Fig. S2) (Shih et al., 2015).

**Fig. 1.**
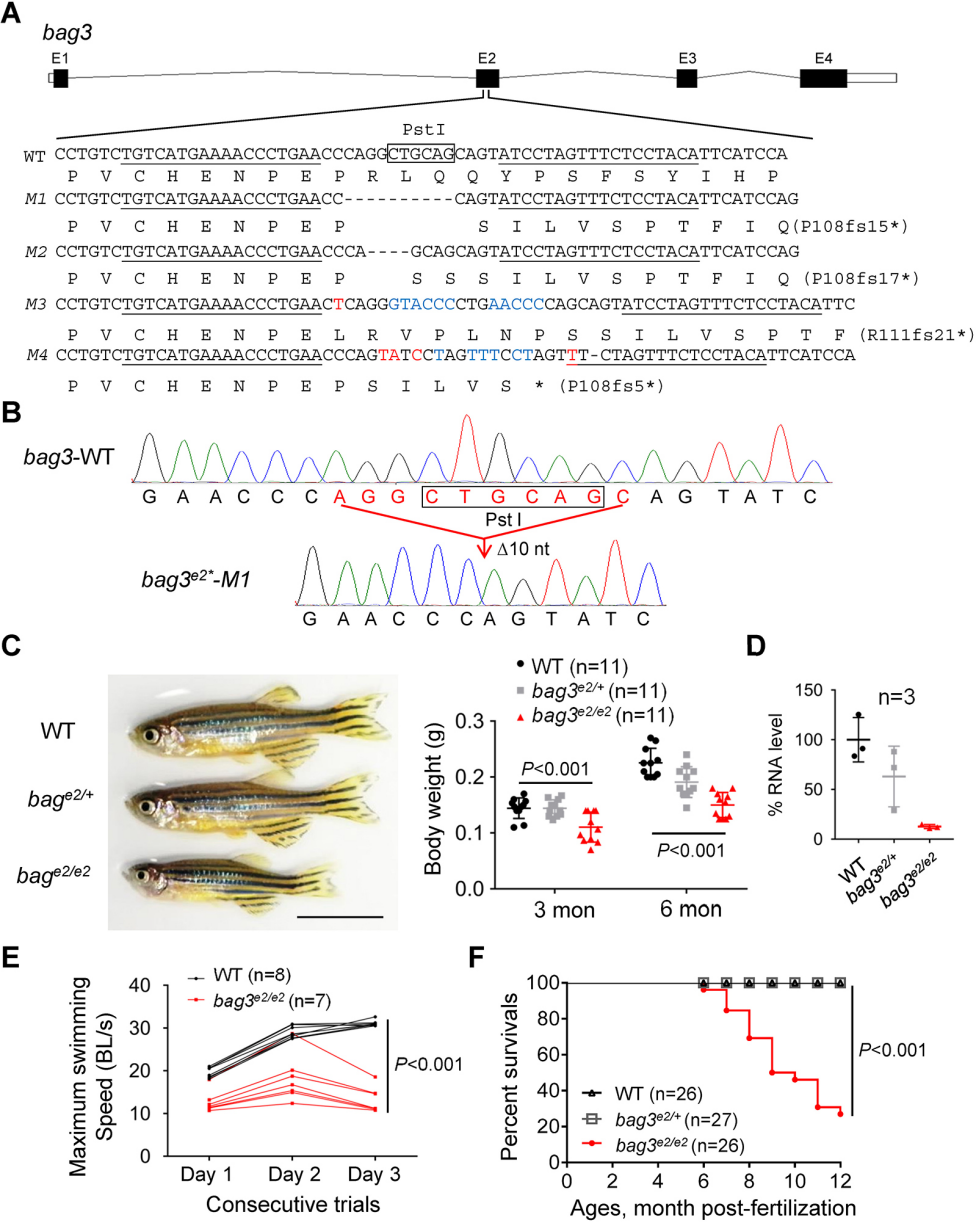
**Generation of zebrafish *bag3* mutants using TALEN.** (A) Schematics of the four *bag3* mutant alleles generated using TALEN. The sequences in the 2nd exon that were targeted by the TALEN pairs are underlined. The PstI restriction enzyme recognition site for genotyping purposes is boxed. Dashed lines indicate deleted nucleotides; nucleotides in blue indicate insertional mutations; nucleotides in red indicate mismatch mutations. Asterisk indicates an early translational stop. fs, frameshift. (B) Chromographs illustrating the sequences of the *bag3* wild-type (*bag3-*WT) and the 10-nucleotide deletion mutant allele (bag3^e2*^-M1). Boxed sequence indicates the restriction enzyme PstI cutting sites in WT that was deleted in the mutant. (C) Representative images and quantification analysis of body length and weight in the *bag3* mutant and WT control at 6 months. Scale bar: 1 cm. *n*=11 animals, one-way ANOVA. (D) Quantitative RT-PCR demonstrated transcript reductions in both the *bag3* heterozygous (*bag3^e2/+^*) and homozygous (*bag3^e2/e2^*) mutants. *n*=3 biological replicates. (E) Maximum swimming speed of the *bag3^e2/e2^* mutant compared with the WT control at 6 months. *n*=7-8, mixed ANOVA. (F) Kaplan–Meier survival curves of *bag3* mutant fish and WT controls. *n*=26-27, log-rank test. Data are mean±s.e.m.

To model *BAG3* cardiomyopathy, we targeted the 2nd exon to generate *bag3* loss-of-function mutants via transcription activator-like effector nuclease (TALEN) technology. Four different truncation alleles predicted to shift the reading frame and lead to a premature stop codon were obtained, designated *bag3^e2^-M1*, *-M2*, *-M3* and *-M4* (Fig. 1A,B and Fig. S3). No evident phenotypes were detected in these mutants during embryonic stages (Fig. S4). However, all four alleles, including both male and female fish, exhibited the same visually noticeable phenotypes, including smaller body size and elongated Meckel's cartilage at 3 months of age (Fig. 1C and Fig. S5). For simplicity, subsequent experiments focused on the *bag3^e2^-M1* allele that harbored a 10-nucleotide deletion, which was renamed *bag3^e2^*. The *bag3* transcripts were reduced 37% in the heart tissues of *bag3* heterozygous fish (*bag3^e2/+^*) and 87% in the homozygous mutant (*bag3^e2/e2^*) (Fig. 1D), likely owing to nonsense-mediated RNA decay. We were unable to assess Bag3 proteins because commercially available antibodies for mammalian BAG3 did not recognize zebrafish Bag3 (data not shown). Swimming capacity was significantly reduced in *bag3^e2/e2^* fish (Fig. 1E). Although it is an important clinical index for heart failure in human patients, reduced swimming capacity in the *bag3^e2/e2^* mutant could also be ascribed to defects in other tissues. Indeed, we noted a skeletal muscle degeneration phenotype in the *bag3^e2/e2^* homozygous mutant at 6 months and in *bag3^e/+^* heterozygous fish at 12 months (Fig. S6). This result is consistent with its function as a myopathy-causative gene in humans (Selcen et al., 2009). The *bag3^e2/e2^* mutant had increased mortality: ∼80% of fish died within 1 year of age (Fig. 1F).

### The *bag3^e2/e2^* mutant manifests reduced cardiac pump function

**Fig. 2.**
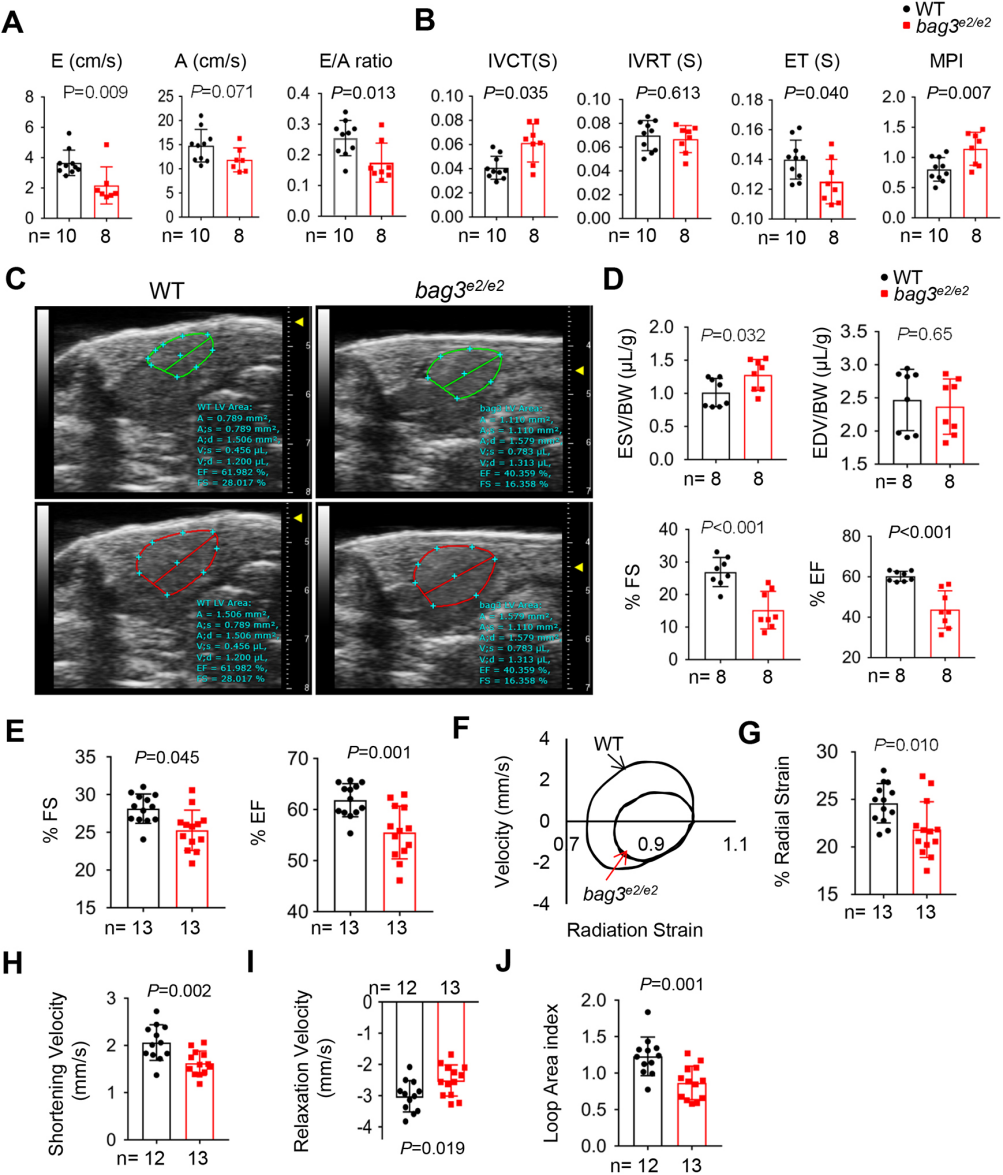
**The *bag3*^e2/e2^ mutant manifests a cardiac dysfunction phenotype.** (A,B) Blood flow indices measured using a custom 30 MHz ultrasound probe. *n*=8-10, Student's *t*-test. (C) Examples of echocardiography images extracted from movies of beating hearts in WT controls and *bag3^e2/e2^* mutants at systole (upper panel) and diastole (lower panel). (D) Quantification of cardiac function indices measured by echocardiography in the *bag3^e2/e2^* mutant and WT control at 6 months. *n*=8, Student's *t*-test. (E-J) Cardiac pump function indices as measured by *ex vivo* heart pump function assay. *n*=12-13, Student's *t*-test. Data are mean±s.e.m.

Next, we focused on evaluating cardiac pump functions in the *bag3^e2/e2^* mutant. We focused our efforts on 6 months of age as the time point at which the fish start to die (Fig. 1F) and presented combined data from both male and female fish because of their similar phenotypes in our initial studies. We initially conducted a pulsed-wave Doppler analysis using the Vevo 2100 imaging system equipped with a 30 MHz transducer (Packard et al., 2017) and detected reduced early (E) ventricular filling velocity, normal late (A) ventricular filling velocity, and a significantly reduced E/A ratio in the *bag3^e2/e2^* mutant, suggesting a decline in diastolic function (Fig. 2A). The *bag3^e2/e2^* fish also had an extended isovolumic contraction time (IVCT) and a reduced ejection time (ET), which led to an overall elevated myocardium performance index (MPI) (Fig. 2B), suggesting a worsening of global cardiac function. More recently, we had access to the Vevo 3100 image system equipped with a 50 MHz transducer (Wang et al., 2017). The higher resolution of this updated system enables the determination of chamber borders of a beating adult zebrafish heart. We detected a significantly reduced ejection fraction (EF) and fractional shortening (FS) (Fig. 2C,D; Movies 1 and 2), which can be largely ascribed to the increased end-systolic volume (ESV)/body weight (BW) ratio. The end-diastole volume (EDV)/BW ratio remained unchanged.

Before we obtained access to the HFE (Vevo 3100) system, we had been developing a Langendorff-like system to quantify cardiac function (Zhang et al., 2018). Driven by both electrical pacing and perfusion via fluid flow through the ventricle, the beating heart could be documented at high resolution using a digital camera *ex vivo*. Consistent with echocardiography-based assays, the indices for pump function and contractility, including EF and FS, were significantly decreased in the *bag3^e2/e2^* mutant, confirming cardiac dysfunction (Fig. 2E; Movie 3). The Langendorff-like *ex vivo* method also enables the measurement of other functional indices, such as the velocities of shortening and relaxation, the primary determinant of myocardial power, and velocity-strain loop area index (an alternative index for the pressure-volume loops to define cardiac performance). All three indices were significantly reduced in the *bag3^e2/e2^* mutant heart (Fig. 2F-J), confirming a contractility dysfunction phenotype.

### The *bag3^e2/e2^* mutant manifests hallmarks of cardiomyopathy

**Fig. 3.**
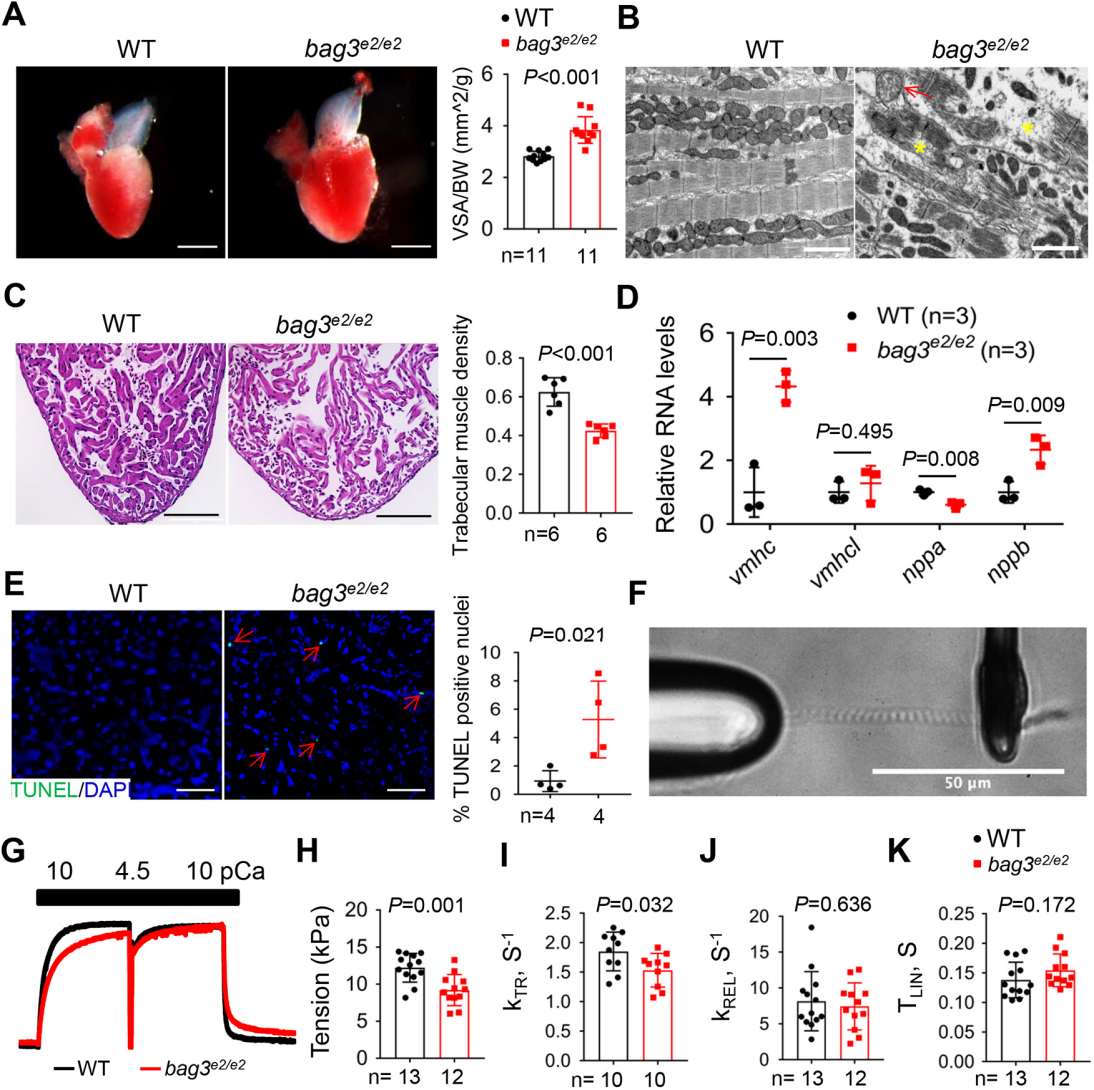
***bag3^e2/e2^* mutants manifest hallmarks of cardiomyopathy resembling DCM in mammals.** (A) Representative images of isolated hearts and quantification of the ventricular surface area (VSA) normalized to body weight (BW) in the *bag3^e2/e2^* mutants and WT controls at 6 months. *n*=11, Student's *t*-test. (B) TEM images confirmed the myofibril degeneration phenotype (yellow asterisks) and identified abnormal mitochondrial swelling (red arrows) in the *bag3^e2/e2^* mutant fish heart at 6 months. (C) Representative images of H&E staining in the apex area and quantification of trabecular muscle density in the *bag3^e2/e2^* mutants and WT controls at 6 months. *n*=6, Student's *t*-test. (D) Quantitative RT-PCR analysis of cardiomyopathy molecular markers in *bag3^e2/e2^* mutant hearts. *n*=3 biological replicates, Student's *t*-test. (E) Representative images of the TUNEL assay and quantification of the percentage of TUNEL-positive nuclei (red arrows) in the *bag3^e2/e2^* mutant and WT control at 6 months. *n*=4, Student's *t*-test. (F) A representative image of a single myofibril isolated from the *bag3^e2/e2^* mutant fish heart attached to glass microtools. (G) Example of a myofibril activation trace (pCa 10→4.5) with force-redevelopment during the release–restretch maneuver and relaxation when pCa was changed back from 4.5 to 10. Activation (pCa=4.5) and the fast release–restretch maneuver were used to measure k_TR_ in myofibrils from WT and the *bag3^e2/e2^* mutant fish heart at 6 months. (H) Quantification of maximal isometric tension in activated single myofibrils. (I-K) Rates of force redevelopment (K_TR_) (I), fast relaxation (K_REL_) (J) and the time of the linear phase of relaxation (T_LIN_) (K) in the *bag3^e2/e2^* mutant and WT control at the single-myofibril level. H-K, *n*=10-13, Student's *t*-test. Data are mean±s.e.m. Scale bars: 1 mm in A; 2 µm in B; 100 µm in C; 20 µm in E; 50 µm in F.

Having defined cardiac pump dysfunction in the *bag3^e2/e2^* mutant, we examined which phenotypic traits of mammalian cardiomyopathy can be recapitulated in the zebrafish model. By quantifying the ventricular surface area (VSA) in dissected hearts, we noted an enlarged heart chamber size, as indicated by the significantly increased VSA/BW index in the *bag3^e2/e2^* mutant at 6 months of age (Fig. 3A; Fig. S8A). Hematoxylin and eosin (H&E) staining revealed myofibril loss and significantly reduced trabecular muscle density in the sectioned *bag3^e2/e2^* fish hearts (Fig. 3C). We confirmed the myofibril degeneration phenotype by transmission electron microscopy (TEM) analysis in 6-month-old *bag3^e2/e2^* fish hearts and noted abnormal mitochondrial swelling and/or vacuolization phenotypes (Fig. 3B). We also noted marginal myofilament disruption and mitochondrial vacuolization phenotypes in the *bag3^e2/+^* heterozygous fish at 12 months but not at 6 months (Fig. S7).

To evaluate *bag3* cardiomyopathy at the cellular and molecular levels, we performed a terminal deoxynucleotidyl transferase-mediated dUTP nick end labeling (TUNEL) assay. We noted significantly increased apoptosis in the *bag3^e2/e2^* mutant at 6 months (Fig. 3E). In contrast, we did not find any significant change in individual cardiomyocyte cell size (Fig. S8B). The proliferation index was not changed, as measured by proliferating cell nuclear antigen (PCNA) staining (Fig. S8C). At the molecular level, we detected aberrant fetal gene reprogramming using quantitative RT-PCR, including elevated *ventricular myosin heavy chain* (*vmhc*, also known as *myh7*) and *natriuretic peptide B* (*nppb*) expression and decreased *natriuretic peptide A* (*nppa*) expression (Fig. 3D).

In contrast to hypertrophic cardiomyopathy (HCM), which often manifests as myofibril hypercontractility, DCM-causative mutations are associated with myofibril hypocontractility (Spudich et al., 2016; Trivedi et al., 2018). Thus, to further define cardiac phenotypes in the *bag3^e2/e2^* mutant fish, we analyzed contractile function at the single-myofibril level (Fig. 3F-K) (Dvornikov et al., 2014). The kinetics of myofibril activation were measured by the activation rate (K_ACT_), which reflects myofibril shortening in response to a fast change in calcium concentration (pCa), and the rate of force redevelopment (K_TR_), which reflects a release–restretch maneuver (Fig. 3G). The myofibril relaxation was measured by the duration of the slow linear phase of relaxation (T_LIN_) and the rate of fast exponential relaxation (K_REL_). We detected significantly reduced maximal isometric tension and K_TR_ in *bag3^e2/e2^* mutant myofibrils (Fig. 3H,I), suggesting hypocontractility of myofibrils, a characteristic associated with sarcomeric DCMs (Chuan et al., 2012; Debold et al., 2007). In contrast, the kinetics of relaxation was not affected (Fig. 3J,K). Next, we measured the intracellular calcium transient in isolated ventricular cardiomyocytes loaded with the Indo-1AM radiometric calcium indicator. We found no significant changes in either the magnitude or kinetics of the Ca^2+^ transients in the *bag3^e2/e2^* mutant heart ventricle (Fig. S9). Together, these comprehensive experiments defined DCM-like phenotypic traits in the *bag3^e2/e2^* mutant.

### Transcriptome analysis identifies mTOR as one of the top signaling pathways altered in the *bag3^e2/e2^* mutant heart

**Fig. 4.**
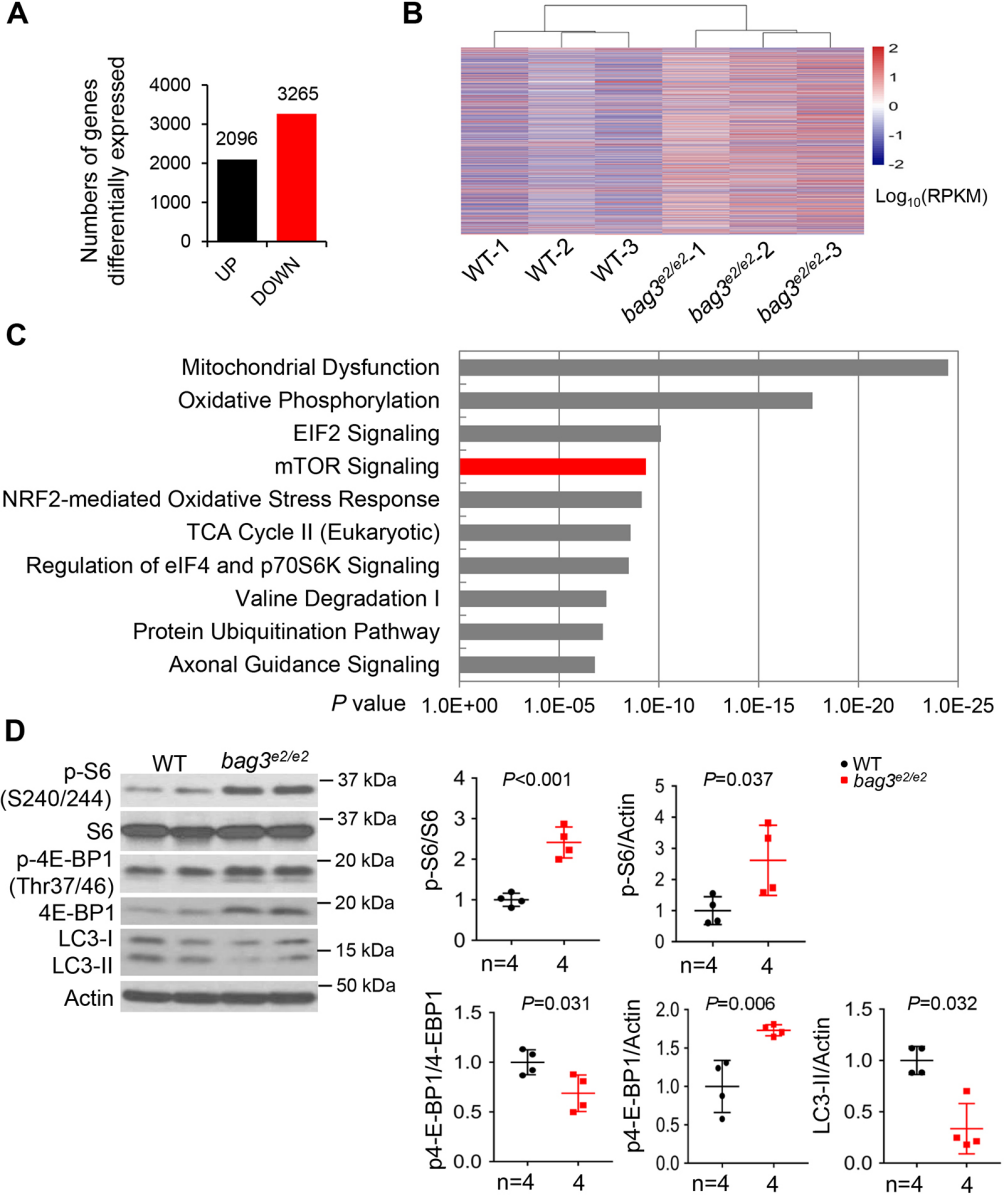
**Transcriptome analysis identifies mTOR as one of the top signaling pathways affected in the *bag3^e2/e2^* mutant.** (A) Numbers of differentially expressed (DE) genes between *bag3^e2/e2^* mutant hearts and WT control with a cutoff of adjusted *P*<0.05. (B) Heat map presenting the expression of 5361 DE genes between the *bag3^e2/e2^* mutant and WT control. Gene expression levels are shown in log_10_ [reads per kilobase per million reads (RPKM)]. (C) The top 10 DE gene enriched pathways with significant *P*-values suggested by IPA. The red column shows that the mTOR pathway was one of the top pathways altered and was subsequently experimentally tested. (D) Representative western blot images and quantification analysis of the expression levels of mTOR downstream proteins. LC3-II protein was examined in heart tissues from fish treated with 50 nM bafilomycin A1 for 4 h. *n*=4 biological replicates, Student's *t*-test. Data are mean±s.e.m.

To elucidate the molecular basis of *bag3* pathogenesis, we carried out transcriptome analysis using 6-month-old fish hearts. Approximately 30 million reads per sample were obtained with three biological repeats, with an average of >92% coverage of the whole annotated zebrafish transcriptome (Zv9). Good reproducibility was observed among independent sample replicates, with >95% Pearson correlation coefficients (Fig. S10). Based on a cut-off of adjusted *P*<0.05, 5361 genes were differentially expressed (DE) in the mutants compared with wild-type (WT) controls (Fig. 4A). A molecular signature analysis of the DE genes distinguished the *bag3^e2/e2^* mutant hearts from the WT control (Fig. 4B). Ingenuity pathway analysis (IPA) ranked DE genes by signaling pathways and identified mitochondrial dysfunction and oxidative phosphorylation as the top two canonical pathways affected, confirming the heart failure phenotypes in the *bag3^e2/e2^* mutant (Fig. 4C). Interestingly, we noted that the genes in the mTOR pathway were ranked the fourth most DE genes. Because mTOR signaling is vital for proteostasis, and mTOR is a known therapeutic target for several types of cardiomyopathies (Sciarretta et al., 2018), we focused on mTOR signaling experimentally. We detected significant hyperphosphorylation of ribosomal S6 protein, a key downstream target of mTOR signaling, in the *bag3^e2/e2^* mutant hearts (Fig. 4D) (Sciarretta et al., 2014). In addition, two negative downstream effectors of mTOR signaling, phosphor-4E-BP1 and LC3-II, were significantly reduced after normalization by total 4E-BP1 or Actin, respectively, as controls. These experiments were carried out in the presence of bafilomycin A1, a specific inhibitor of the autophagosome-lysosome, to reveal autophagic flux and static autophagy. Together, our unbiased transcriptome analysis and subsequent experimental validation suggested mTOR activation in the *bag3^e2/e2^* mutant.

### *mtor^xu015+/−^* mitigates *bag3* cardiomyopathy

**Fig. 5.**
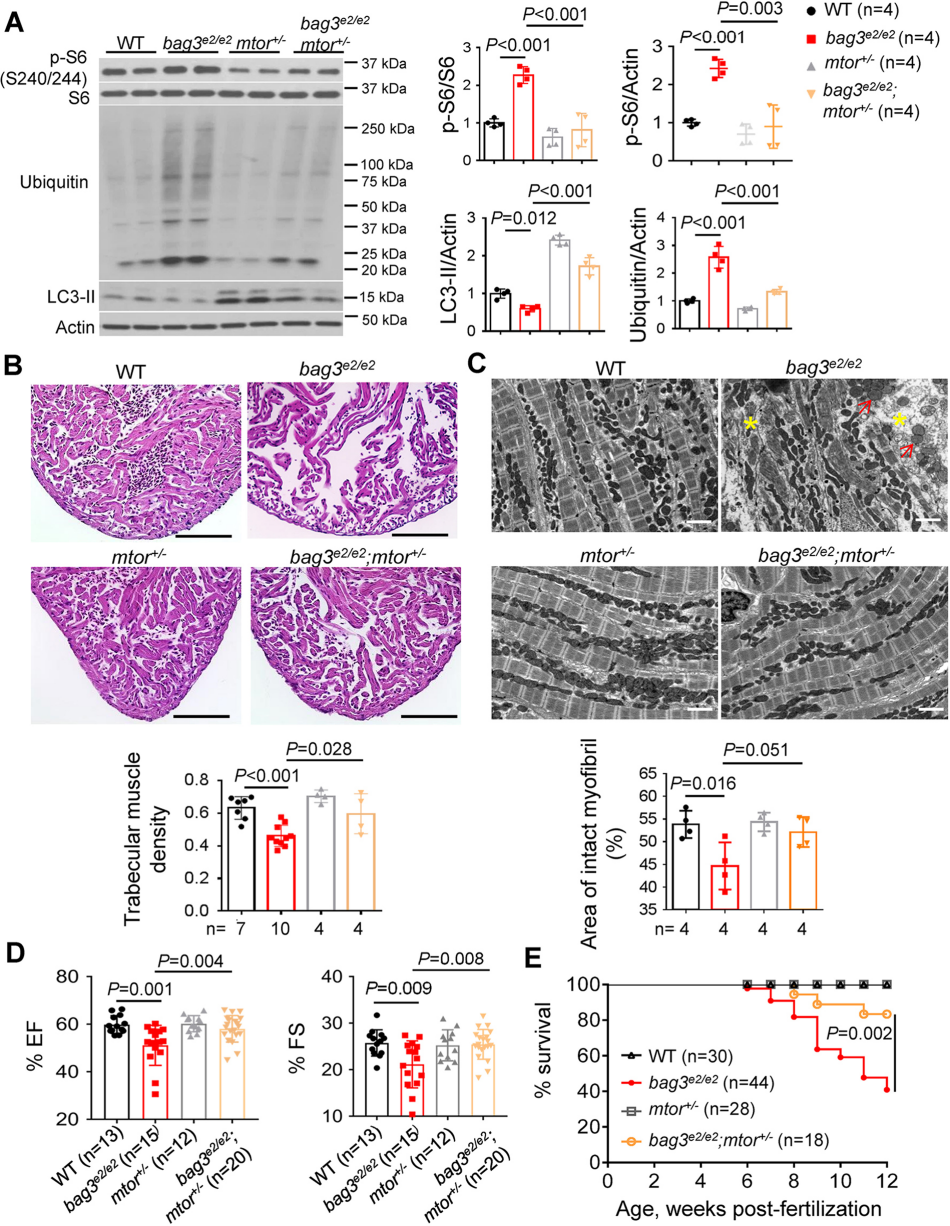
**Genetic testing reveals the therapeutic effects of *mtor^+/−^* on *bag3* cardiomyopathy.** (A) Western blot and quantification analysis of ribosomal S6 protein (p-S6), LC3-II and ubiquitinated proteins in the *bag3^e2/e2^;mtor^+/−^* double mutants compared with their corresponding single mutants and the WT control at 6 months. LC3-II protein was examined in heart tissues dissected from fish treated with 50 nM bafilomycin A1 for 4 h. *n*=4 biological replicates, one-way ANOVA. (B) Representative H&E staining images from the apex area and quantification of the trabecular muscle density from the *bag3^e2/e2^;mtor^+/−^* double mutants and their corresponding single mutants and WT controls at 6 months. *n*=4-10, one-way ANOVA. (C) TEM images and quantification analysis of *bag3^e2/e2^;mtor^+/−^* mutants at 6 months compared with their siblings harboring either single mutants or the WT control. Yellow asterisks indicate regions of myofibril degeneration. Red arrows indicate mitochondria with abnormal swelling. (D) Percent EF and FS of *bag3^e2/e2^*;*mtor^+/−^* double mutant fish at 6 months compared with their corresponding single mutants and the WT control. *n*=12-20, one-way ANOVA. (E) Kaplan–Meier survival curves of the *bag3^e2/e2^;mtor^+/−^* double mutants compared with their corresponding single mutants and the WT control*. n*=18-44, log-rank test. Scale bars: 100 µm in B; 2 µm in C. Data are mean±s.e.m.

Last, we tested whether genetic inhibition of mTOR is cardioprotective for *bag3* cardiomyopathy. *mtor^xu015^*, a hypomorphic zebrafish mutant (Ding et al., 2011), was bred into the *bag3^e2/e2^* mutant to generate *bag3^e2/+^;mtor^xu015+/−^* mutants, which were sequentially incrossed to generate *bag3^e2/e2^;mtor^xu015+/−^* double mutants. By analyzing these double mutants, compared with their siblings harboring either a single mutant or WT, we found that partial mTOR inhibition through the *mtor^xu015+/−^* mutant largely normalized the hyperphosphorylation of ribosomal S6, rescued the impaired autophagic flux as revealed by the restored LC3-II protein, and reduced the ubiquitinated protein aggregations in the *bag3^e2/e2^* mutant (Fig. 5A). The cardiomyopathy hallmarks present in the *bag3^e2/e2^* mutant, including myofibril loss, myofibril degeneration and mitochondrial swelling, were all largely rescued in the *bag3^e2/e2^;mtor^xu015+/−^* double mutants (Fig. 5B,C). Importantly, the introduction of the *mtor^xu015^* allele into *bag3^e2/e2^* fish significantly restored cardiac pump function, as measured using an *ex vivo* assay (Fig. 5D). As a consequence, fish survival was significantly improved (Fig. 5E). Together, these data provide strong genetic evidence to support *mtor* as a candidate therapeutic target for *bag3* cardiomyopathy in zebrafish.

## DISCUSSION

### The *bag3^e2/e2^* mutant is likely a DCM model in adult zebrafish

In this study, we report the generation of *bag3^e2/e2^* mutants in adult zebrafish to model *BAG3* cardiomyopathy. The reading-frame-shift genotype of *bag3^e2/e2^* mutants would most likely confer a loss of function, recapitulating genetic lesions in mammals that lead to *BAG3* cardiomyopathy (Fang et al., 2017; Homma et al., 2006). The *bag3^e2/e2^* mutant fish developed hallmarks of mammalian cardiomyopathy at 6 months, including reduced cardiac pump function, increased ventricular chamber size, myofibril degeneration, abnormal mitochondria, reduced exercise capacity and aberrant expression of fetal genes that are molecular markers of cardiomyopathy (Harvey and Leinwand, 2011; Taegtmeyer et al., 2010). Despite the robust expression of *bag3* during the early embryonic stage, no evident cardiac or somite phenotypes were detected, suggesting that developmental defects are less likely the root cause of cardiac phenotypes at 6 months. Similar to DCM in mammals, which is characterized by ventricular chamber enlargement, systolic dysfunction and myofibril hypocontractility (Merlo et al., 2018), the following three phenotypic traits in the *bag3^e2/e2^* mutants further suggest a DCM-like model in zebrafish. First, at the cardiac systolic function level, we detected reduced EF that was ascribed to increased ESV/BW, resembling the eccentric hypertrophy that is a characteristic of DCM in mammals (Japp et al., 2016; McNally and Mestroni, 2017; Merlo et al., 2018). Second, at the single-myofibril level, we detected decreased maximal isometric tension and the reduced activation of myofibril kinetics, suggesting ‘hypocontractility’, another key feature of DCM hearts in mammals (Spudich et al., 2016). Third, we detected ventricular chamber enlargement, as indicated by the increased VSA/BW, which can serve as a surrogate DCM index for the unchanged EDV from our HFE analysis. Notably, both reduced ventricular systolic function and enlarged ventricular chamber size have been reported in the *ttna^tv/+^* model (Huttner et al., 2018). Whether the *ttna^tv/+^* model also manifests ‘hypocontractility’ at the single-myofibril level remains to be examined.

Because human *BAG3* cardiomyopathy is an autosomal dominant disease, we acknowledge the limitation of using a homozygous *bag3^e2/e2^* mutant to model this subtype DCM. As exemplified by a recent study of *Bag3* knockouts in mice (Fang et al., 2017), homozygous *Bag3* mutants can be a highly efficient surrogate for mechanistic studies, as long as the key phenotypic traits can be recapitulated. As a future direction, much milder phenotypes in the aged *bag3^e2/+^* heterozygous mutant will be analyzed in more detail, which might be a more faithful model recapitulating human *BAG3* cardiomyopathy. This effort is feasible in zebrafish because we were able to detect cardiac phenotypes in *bag3^e2/+^* heterozygous fish at 12 months. Notably, caution must be taken when interpreting cardiac phenotypes in these compound knockout mutants because the noncardiac expression of *bag3*, such as skeletal muscle, could indirectly affect cardiac-related indices, such as reduced swimming capacity phenotype and increased mortality. We also acknowledge the limitations of using zebrafish as a lower vertebrate to model DCM. For example, ventricular wall thickness, a major phenotypic trait that discerns DCM from HCM, is difficult to measure in a highly trabeculated zebrafish heart (Wang et al., 2011). We suggest that the reduced trabecular muscle density in the *bag3^e2/e2^* mutant heart might serve as a surrogate index of thinner walls in mammals. In addition, certain phenotypes in mammalian *Bag3* DCM models, such as a reduced Ca^2+^ response (Feldman et al., 2016), cannot be recapitulated in the zebrafish *bag3* model. These intrinsic weaknesses need to be considered when developing therapies for human diseases using this new vertebrate model.

### Integration of new phenotyping tools enables the discernment of different subtypes of cardiomyopathies in adult zebrafish

The paucity of cardiac phenotyping tools in adult zebrafish with a small heart size has been a major hurdle that curtails the surgical power offered by this efficient vertebrate model. Here, we integrated new cardiac phenotyping tools developed recently and demonstrated that enriched cardiac function details can be obtained, enabling the discrimination of different DCM subtypes. For example, by employing ultrasound-based Doppler imaging (Packard et al., 2017), we detected prolonged IVCT and normal isovolumic relaxation time (IVRT) in our *bag3^e2/e2^* mutant. In contrast, prolonged IVRT was reported in the *ttna^tv/+^* model (Huttner et al., 2018), but IVCT was not reported. We also noted significantly reduced E, unchanged A, and as a consequence, a reduced E/A ratio in our *bag3^e2/e2^* mutant. In contrast, only reduced A was noted in male fish of the *ttna^tv/+^* model, whereas both the E and E/A ratios remained unchanged. Moreover, increased E and E/A ratios were reported in the DIC model (Packard et al., 2017), which is different from both the *bag3^e2/e2^* and *ttna^tv/+^* models.

The improved phenotyping tools justify future efforts to compare more cardiomyopathy models in zebrafish. Additional inherited zebrafish models harboring a different DCM-causative gene and/or a known HCM-causative gene should be generated. Common phenotypes between the *bag3^e2/e2^* mutant and other DCM models and/or different phenotypes between DCM and HCM models will lead to optimized guidelines to define cardiomyopathies of different etiologies. On the other hand, different phenotypes among different subtypes of DCM will justify this simple vertebrate model for individualized medicine for cardiomyopathies.

### The zebrafish *bag3* cardiomyopathy model can be used to search for therapeutic targets, such as *mtor*

The improved phenotyping tools enabled us to determine the effects of therapy on the *bag3^e2/e2^* cardiomyopathy model more reliably. Through a combination of unbiased RNA-seq analysis with targeted genetic validation, we found the activation of mTOR signaling in the *bag3* mutant and showed the therapeutic capacity of the genetic inhibition of mTOR signaling. Mechanistically, we noted that both impaired autophagic flux and elevated ubiquitinated protein aggregation in the *bag3^e2/e2^* mutant hearts can be largely rescued by the genetic inhibition of mTOR, suggesting that the therapeutic effects of mTOR inhibition are ascribed to repairing the primary damage of protein homeostasis caused by *bag3* deficiency. Consistent with this hypothesis, a recent biochemical and cell culture study showed that Bag3 balances protein synthesis and degradation by spatially regulating mTOR complex 1 (mTORC1) via direct interaction with tuberous sclerosis protein 1 (TSC1) (Kathage et al., 2017).

On the other hand, it is also possible that mTOR inhibition attenuates a common pathological event that is shared among different types of cardiomyopathy, as suggested by its cardioprotective effects on several subtypes of cardiomyopathies in both zebrafish and mouse (Ding et al., 2011; Sciarretta et al., 2018). Consistent with this hypothesis, impaired cardiomyocyte autophagy was reported in rat models of Titin-truncating variants (TTNtv), which can be rescued by rapamycin, a specific pharmacologic inhibitor of mTOR signaling (Zhou et al., 2019). Dysregulated proteostasis might be part of the common pathological event, and the accumulation of toxic proteins might further drive the pathogenesis into irreversible heart failure. Further mechanistic studies and drug development efforts of mTOR-based therapy are needed, which could benefit a broad spectrum of cardiomyopathies with different etiologies.

In summary, this study established the *bag3^e2/e2^* mutant as a DCM model in adult zebrafish and demonstrated the feasibility of identifying therapeutic strategies, such as the genetic inhibition of mTOR signaling. To fully leverage the potential of zebrafish as a new animal model, one future research direction is to conduct mutagenesis screens for systematically identifying gene modifiers and therapeutic strategies. The feasibility of such a forward-genetic strategy has been recently demonstrated for the DIC model (Ding et al., 2016). It will be interesting to explore whether the genetic modifiers and related therapeutic targets identified from the DIC model can be extrapolated to an inherited cardiomyopathy model, such as *bag3^e2/e2^*.

## MATERIALS AND METHODS

### Experimental animals

Zebrafish (*Danio rerio*; WIK strain) were maintained under a 14 h light/10 h dark cycle at 28.5°C and handled with care. All animal study procedures were performed in accordance with the Guide for the Care and Use of Laboratory Animals published by the US National Institutes of Health (NIH Publication No. 85-23, revised 1996). The anesthetic agent used in the study is tricaine (0.02%) (Argent Chemical Laboratories), through incubation in fish system water for 5-10 min. Overdose of a chemical was used as the means of euthanasia for the zebrafish: 1% 3-aminobenzoic acid ethyl ester (Tricaine S) was added to the water as the final concentration. Animal study protocols were approved by the Mayo Clinic Institutional Animal Care and Use Committee (IACUC), protocol number A3531.

### Generation of *bag3* mutants via TALEN

TALEN techniques were employed to generate *bag3* mutants, according to our previously published approaches (Shih et al., 2016). Briefly, TALEN primer pairs targeting the 2nd exon of the *bag3* gene were designed using Zifit (http://zifit.partners.org/ZiFiT/ChoiceMenu.aspx). The TALEN left-arm binding sequence 5′-TGTCATGAAAACCCTGAA-3′ and right-arm binding sequence 5′-ATCCTAGTTTCTCCTACA-3′ were used. Both TALENs were then assembled using a Golden Gate kit (Addgene) (Cermak et al., 2011). Capped mRNAs were synthesized using a mMESSAGE mMachine T3 kit (Ambion). Approximately 25 pg capped mRNA was injected into one-cell-stage embryos. Founder fish (F0) were raised to adulthood and outcrossed to generate F1 embryos. Individual F1 embryos were used for genotyping PCR to identify mutant alleles (forward primer: 5′-CGGCGTATAAAGAATTGCTGG-3′; reverse primer: 5′-GTGAAGTAGGTGAGCAAGAC-3′). The resulting PCR products were digested with the restriction enzyme PstI to identify the WT or mutant genotype. The uncut PCR products were Sanger-sequenced to determine the precise genomic lesions. Four different *bag3* mutant alleles that presumably resulted in different shifts of the reading frame for each mutant locus were selected for continuous outcrosses up to the 5th generation and subsequent phenotypic analysis.

### Cardiac functional phenotyping via Doppler imaging

The initial cardiac phenotypic analysis was performed on *bag3* mutants using our reported Doppler imaging technique (Packard et al., 2017). Briefly, adult zebrafish at designated stages were anesthetized in tricaine (0.02%) for 10 min and placed ventral side facing upward. A 30 MHz transducer was placed ∼6 mm above the ventral side of the zebrafish vertically to acquire an ultrasound signal. Under the guidance of B-mode imaging, a Doppler gate (window) was positioned downstream from the atrioventricular (AV) valve in the ventricular inflow region to interrogate inflow velocities. The pulse repetition frequency (PRF) for pulsed-wave (PW) Doppler was set to 9.5 kHz, and the estimated Doppler angle was ∼0° because the blood flow of the zebrafish cardiac chambers is in the dorsal-ventral direction. PW Doppler signals were recorded in the control and doxorubicin groups for ∼3 s and stored for offline analysis using MATLAB. To interrogate cardiac hemodynamics, we analyzed Pulse Wave Doppler signals of passive (E wave velocity) and active (A wave velocity) ventricular filling during diastole. These measures – IVCT, ET across the ventriculobulbar valve, and IVRT – determined the MPI as follows: MPI=(IVCT+IVRT)/ET. The MPI thus constitutes an integrated measure of systolic and diastolic function, with increases in MPI values indicating a worsening of cardiac function.

### Cardiac functional phenotyping via the Vevo 3100 echocardiography system

Cardiac functional phenotypes were subsequently measured and analyzed using the Vevo 3100 high-frequency imaging system equipped with a 50 MHz linear array transducer (Fujifilm VisualSonics). Acoustic gel (Aquasonic^®^ 100, Parker Laboratories) was applied over the surface of the transducer to provide adequate coupling with the tissue interface. Adult zebrafish at 6 months old were anesthetized in tricaine (0.02%) for 5 min, placed ventral side up, and held in place with a soft sponge stage. The 50 MHz (MX700) transducer was placed above the zebrafish to provide a sagittal imaging plane of the heart. B-mode images were acquired with an imaging field of view of 9.00 mm in the axial direction and 5.73 mm in the lateral direction, a frame rate of 123 Hz, medium persistence and a transmit focus at the center of the heart. Image quantification was performed using the VevoLAB workstation. Data were acquired and processed according to a recent report (Wang et al., 2017). Ventricular chamber dimensions were measured from B-mode images using the following two indices: FS=(EDD-ESD)/EDD and fractional area change (FAC)=(EDA-ESA)/EDA. EDD and ESD are the perpendicular distances from the ventricular apex to the ventricular basal line at the end-diastolic stage and end-systolic stage, respectively; EDA and ESA are defined as the areas of the ventricular chamber at the end-diastolic stage and end-systolic stage, respectively. For each index in individual fish, measurements were performed on three to five independent cardiac cycles to acquire average values.

### Cardiac functional phenotyping via an *ex vivo* assay

Cardiac functional phenotypes were also assessed using our recently developed *ex vivo* assay (Zhang et al., 2018). Briefly, at the designated stages, adult zebrafish hearts were isolated, cannulated by 34-G ultrathin catheters through the atrioventricular canal under a stereo-microscope (Leica M165C), perfused with Ca^2+^-containing Tyrode solution [132 mM NaCl, 2.5 mM KCl, 4 mM NaHCO_3_, 0.33 mM NaH_2_PO_4_, 1 mM CaCl_2_, 1.6 mM MgCl_2_, 10 mM HEPES, 5 mM glucose and 5 mM sodium pyruvate (pH 7.5)] using a peristaltic pump EP-1 Econo Pump (Bio-Rad), and paced using a stimulator MyoPacer (Ionoptix; ∼15 V, 10 ms, 2 Hz). Movies of paced heart beats were recorded using a 14-megapixel Amscope MU1403 camera (66 fps). Images in the perpendicular plane of the heart were also obtained using a 45-degree right angle aluminum mirror (Thorlabs). The EDV and ESV were calculated using the biplane area-length formula: V= 2/3A_AL_×L_AL_, where A_AL_ denotes the ventricle area in the transverse plane (short axis), L_AL_ denotes the ventricle length in the longitudinal plane (long axis), E=(EDV–ESV)/EDV. FS=(L_d_–L_s_)/L_d_, where L_d_ and L_s_ denote the ventricle lengths at diastole and systole, respectively. Three cardiac cycles were analyzed and averaged to obtain each parameter. Image series were analyzed using ImageJ software (NIH) and a custom-written MATLAB code. The code performed edge detection of all four heart images (two hearts, two planes); then, radial strain was measured by averaging the number of ventricle radii (set by hand; from center of mass to periphery) on a frame-to-frame basis. In three cardiac cycles, we analyzed this radial strain and the maximal contraction and relaxation velocities (by differentiation of the deformation parameter and finding the extrema of the function). We plotted velocity against strain, connecting points frame-to-frame to obtain a velocity-strain loop that was a good representation of cardiac contractility.

### Single-myofibril assays

Cardiac phenotypes were further defined at the single-myofibril level. Single myofibrils were prepared as previously described (de Tombe and Stienen, 2007; Dvornikov et al., 2014; Walker et al., 2011). Briefly, hearts were permeabilized (skinned) in 1% (v/v) Triton X-100 in a relaxing buffer supplemented with protease inhibitors at 4°C overnight, followed by homogenization in ice-cold relaxing solution at 20,000 rpm for 10 s (homogenizer MDT500, 5 mm probe, MicroDisTec). The pellet was then resuspended and placed into the tissue bath for subsequent mounting to fire-polished glass microtools in relaxing solution containing 10 mM EGTA. The left probe was stiff for fiber stretching; the right probe was compliant, serving as a force transducer. The tip of the right probe was ∼1 µm, and its stiffness was calibrated to be 10-50 nN/µm. The attached myofibril was then superfused with alternating solution streams of relaxing and activating solutions (1 mM EGTA) emanating from a double-barreled pipette (∼180 µl/min) that was mounted on a translation stage capable of rapid (<5 ms) solution switches.

The activation of the myofibril led to its shortening and, therefore, displacement of the right probe. The positions of the left and right probes and the length of the sarcomere of a single myofibril were detected optically with a fast camera (Teledyne Dalsa Genie HM640) and HVSL software (Aurora Scientific). The release–restretch maneuver (release is ∼20% of the fiber length) was performed using a piezo controller (Thorlabs) driving the left probe. All motors, the perfusion system (Warner Instruments) and recordings were fully controlled by custom-written Labview software (courtesy of Pieter de Tombe, Loyola University Chicago, IL, USA). A sarcomere length of 2.0 µm was used for force-pCa experiments. To generate the force-pCa curve, myofibrils were subjected to various pCa-activating buffers (in a range of 5.5-6). The passive stiffness test was performed by releasing the fiber at gradually increasing sarcomere/fiber lengths (SL) (from slack position to SL=2.4 µm). This approach allows the steady-state [Ca^2+^] saturated force and the rates of Ca^2+^-induced force activation and relaxation to be measured. Experiments were performed at SL=2.3 µm. Rates of activation (k*_Ca_*), rapid release–restretch force redevelopment (k*_tr_*), and biphasic relaxation (k*_lin_* and k*_exp_*), together with the duration of the slow linear relaxation phase (T*_lin_*), were analyzed by linear and exponential curve fitting using offline custom in-house written software (Labview). The compositions of the bath, relaxing and activating solutions were as previously described (Dvornikov et al., 2014). The exclusion criterion for fibers was a release of the developed tension of more than 20% per set of contractions. All zebrafish single-myofibril mechanics experiments were performed at 10°C.

### Single-cardiomyocyte cell size measurements

Cardiomyocytes from dissected ventricles of *bag3^e2/e2^* mutants and WT control fish were dissociated as reported (Warren et al., 2001). Dissociated cardiomyocytes were then resuspended in L-15 medium containing 10% fetal bovine serum (Invitrogen) and placed in Lab-Tek eight-well chambers (Thermo Fisher Scientific). Healthy dissociated cardiomyocytes were usually attached to the chamber within 1 h, and the attached cardiomyocytes were then cultured at 28.5°C for 12 h, followed by α-actinin antibody immunostaining to confirm their cardiomyocyte identity (Yang and Xu, 2012a). Images of α-actinin-stained cardiomyocytes were captured, and the cardiomyocyte area was measured by outlining each individual cardiomyocyte using ImageJ software.

### Measurement of ventricular surface-area-to-body weight index

Owing to the small size of an adult zebrafish heart, we previously calculated VSA normalized by BW as an index to assess heart size in adult zebrafish (Ding et al., 2011). To measure VSA, individual zebrafish hearts were dissected and imaged next to a millimeter ruler under a Leica MZ FLI III microscope. The largest projection of a ventricle was outlined using ImageJ software. To measure BW, fish were anesthetized in tricaine (0.02%) solution for 3 min, semidried on a paper towel and weighed on a scale. VSA/BW was then determined by the largest projection area of the ventricle (in mm^2^) divided by body weight (in g).

### Swimming tunnel assay

The swimming tunnel assay was conducted using a swim tunnel respirometer (Mini Swim 170, Loligo Systems) to measure the swimming capacity of *bag3* mutants. This protocol was derived from previous reports with modifications (Sun et al., 2015; Wang et al., 2011). Briefly, *bag3* heterozygous and homozygous mutants were raised together with age-matched WT controls. All fish were fasted for 24 h before the first swimming capacity measurement. To evaluate swimming capacity, adult fish were transferred, four to eight fish per group, into the swim tunnel respirometer with an initial water speed of 9 cm/s for a 20-min acclimation. Water flow was then increased in stages of 8.66 cm/s (Ti) every 150 s (Tii) until all fish were exhausted. The speeds at the last stage (Uii) and the previous stage (Ui) were recorded for each individual fish. The critical swimming capacity (Ucrit) was calculated with the following formula: Ucrit=Ui+[Uii×(Ti/Tii)]. Ucrit was then normalized to the body length (BL) of the corresponding individual. The same batches of fish were tested at 48 h and 96 h later for validation.

### Western blotting

Western blotting was performed as described previously (Ding et al., 2016). Embryos from 6 days postfertilization (dpf) or fresh hearts isolated from adult fish were transferred immediately to RIPA buffer (Sigma-Aldrich) supplemented with complete protease inhibitor cocktail (Roche) and homogenized using a Bullet Blender tissue homogenizer (Next Advance). The resultant protein lysates were subjected to western blotting using a standard protocol. The following primary antibodies were used: anti-actin (1:8000, Santa Cruz Biotechnology, sc-1615), anti-phospho-mTOR (Ser2448) (1:2000, Cell Signaling Technology, 2971), anti-phospho-S6 ribosomal protein (Ser240/244) (1:5000, Cell Signaling Technology, 2215), anti-S6 ribosomal protein (1:8000, Cell Signaling Technology, 2217), anti-phospho-4E-BP1 (Thr37/46) (1:1000, Cell Signaling Technology, 2855), anti-4E-BP1 (1:2000, Cell Signaling Technology, 9644), anti-ubiquitin (1:1000, Thermo Fisher Scientific, PA5-17067) and anti-LC3 (1:3000, Novus Biologicals, NBP100-2331).

### Histology

Embryos at 6 dpf, heart and somite tissues were harvested from adult fish at the designated stages after euthanization by incubation with 0.032% tricaine for 10 min. Embryonic somite or dissected tissues were immediately fixed in 4% PBS-buffered formaldehyde and sent to the Mayo Clinic Histology Core Laboratory for subsequent sample processing and H&E staining. Images for somite tissues at 6 dpf, and heart tissues from the apex region were captured using the EVOS FL Auto Imaging System (Thermo Fisher Scientific). The density of the trabecular muscle was quantified using ImageJ software. For PCNA immunostaining, 8 µm cryostat-cut frozen sections (Leica CM3050S) were subjected to immunostaining using previously described methods (Yang and Xu, 2012b). The primary anti-PCNA antibody (1:3000, Sigma-Aldrich, p8825) was used. For the TUNEL assay, cryostat-sectioned fish ventricles (8 μm) were stained with the *In-Situ* Cell Death Detection Kit, Fluorescein (Roche Applied Science) according to the manufacturer's protocol. Both the anti-PCNA antibody and TUNEL-stained tissue samples were mounted using Vectashield mounting medium with DAPI (Vector Laboratories, H-1200). All images were captured using a Zeiss Axioplan II microscope equipped with ApoTome and AxioVision software (Carl Zeiss Microscopy).

### Transmission electron microscopy

For TEM analysis, a single zebrafish adult heart was dissected and fixed immediately in Trump's fixative solution [4% paraformaldehyde and 1% glutaraldehyde in 0.1M phosphate buffer (pH 7.2)] at room temperature for 1 h, followed by overnight incubation at 4°C. The fixed samples were subsequently processed and imaged at the Mayo Clinic Electron Microscopy Core Facility using a Philips CM10 transmission electron microscope. Quantification of the area of intact myofibril was largely performed based on a published report (Huttner et al., 2018). Briefly, TEM images from 2500- to 8000-fold magnification from three areas, including the apex, anterior wall near the base/bulbo-aortic valve and posterior wall near the AV-valve, were captured. The area of intact myofibril from each image was outlined and expressed to the total area of the image using ImageJ.

### RNA-seq data collection and analysis

Total RNA was extracted from dissected ventricular tissue of 6-month-old *bag3^e2/e2^* mutants and WT siblings. Five ventricles were pooled as one sample. Six total samples (three biological replicates for each genotype) were sequenced using the HiSeq 2000 platform (Illumina) with a 50-bp paired-end sequencing protocol in the Mayo Clinic DNA Sequencing Core Facility. Raw RNA-seq reads for each sample were aligned with TopHat (Version 2.0.12) (Kim et al., 2013) to the zebrafish genome assembly (Zv9) using the Ensembl annotation Zv9 (Danio_rerio.Zv9.79.gtf). Each gene was assembled with Cufflinks (Version 2.2.1) (Trapnell et al., 2010). Genes were considered to be differentially expressed across different groups if they exhibited a greater than twofold change and a false discovery rate of less than 0.05 according to the Cuffdiff script from Cufflinks. Unsupervised hierarchical clustering was performed with Pearson correlation and scaled based on the fragments per kilobase of transcript per million mapped reads value using the pheatmap R package (https://github.com/raivokolde/pheatmap). The gene lists of interest were annotated by IPA (Qiagen) (http://www.ingenuity.com/). We queried the IPA with the gene list of interest to map and generate putative biological processes/functions, networks and pathways based on the manually curated knowledge database of molecular interactions extracted from the public literature. The enriched pathways and gene networks were generated using both direct and indirect relationships/connectivity. These pathways and networks were ranked by their enrichment score, which measures the probability that the genes were included in a network by chance. Both the primary RNA-seq raw and processed datasets have been deposited in GEO under accession number GSE135823.

### Statistics

Survival curve, echocardiography and *ex vivo* heart function analyses are from cumulative data. The unpaired two-tailed Student's *t*-test was used to compare two groups. One-way or mixed ANOVA was used to assess differences among multiple groups, as appropriate. The log-rank test was used to determine the difference in animal survival. All quantitative data are presented as the mean±s.e.m. Sample size (*n*) represents animal number, otherwise specifically designated as biological replicates. *P*-values <0.05 were considered to be significant. All statistical analyses were performed using GraphPad Prism 7 and/or R Statistical Software Version 3.6.1. For the post hoc analysis, we employed Tukey's test to confirm our findings.

## Supplementary Material

Supplementary information
