# Supplementary material for: Haploinsufficiency of mechanistic target of rapamycin ameliorates bag3 cardiomyopathy in adult zebrafish
Source: Dis Model Mech. 2019 Oct 1;12(10):dmm040154. doi: 10.1242/dmm.040154 (PMC6826022; doi:10.1242/dmm.040154)
Supplement: Supplementary information [file dmm-12-040154-s1.pdf]

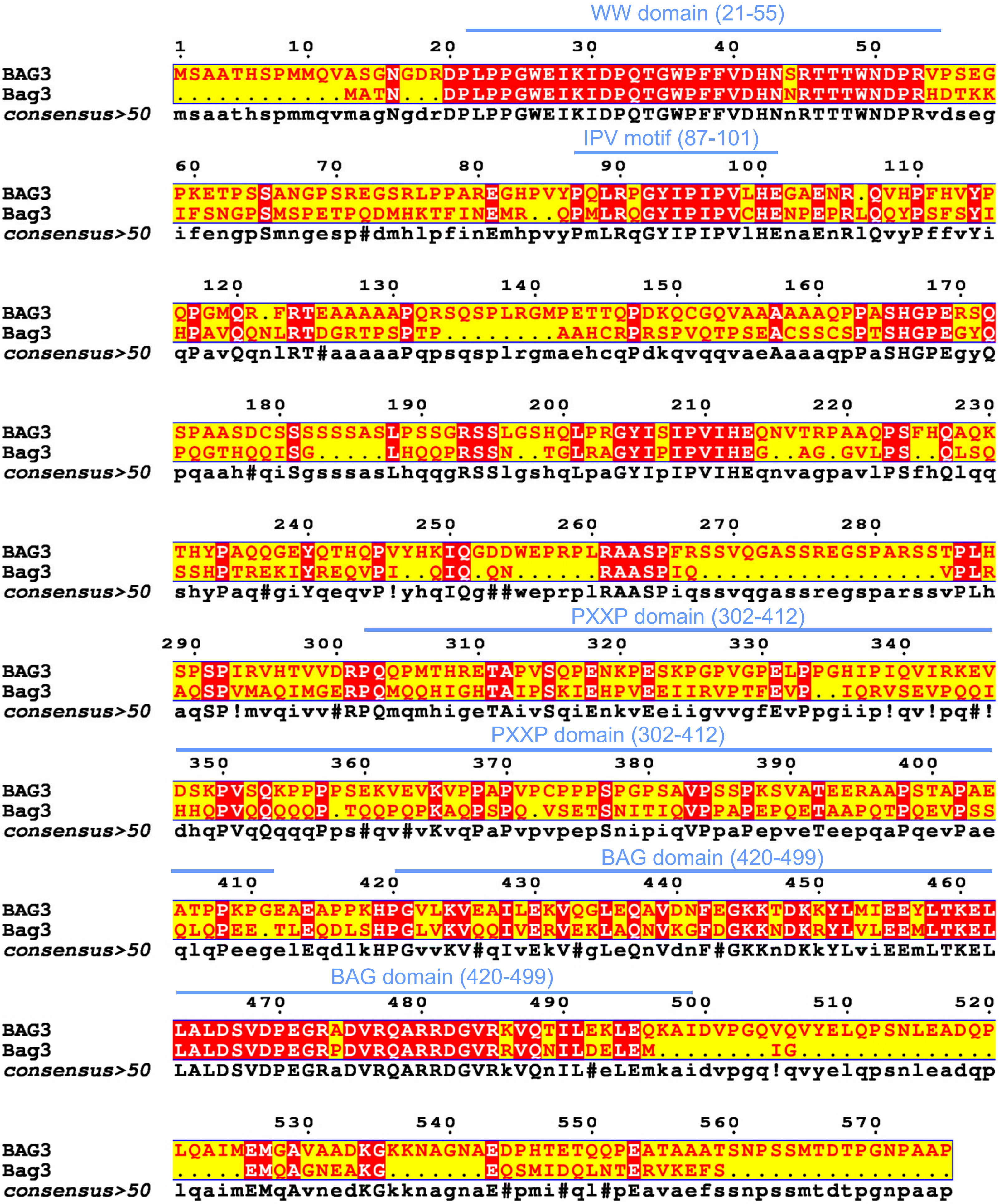

Fig. S1. Alignment of the human BAG3 and zebrafish Bag3 proteins.

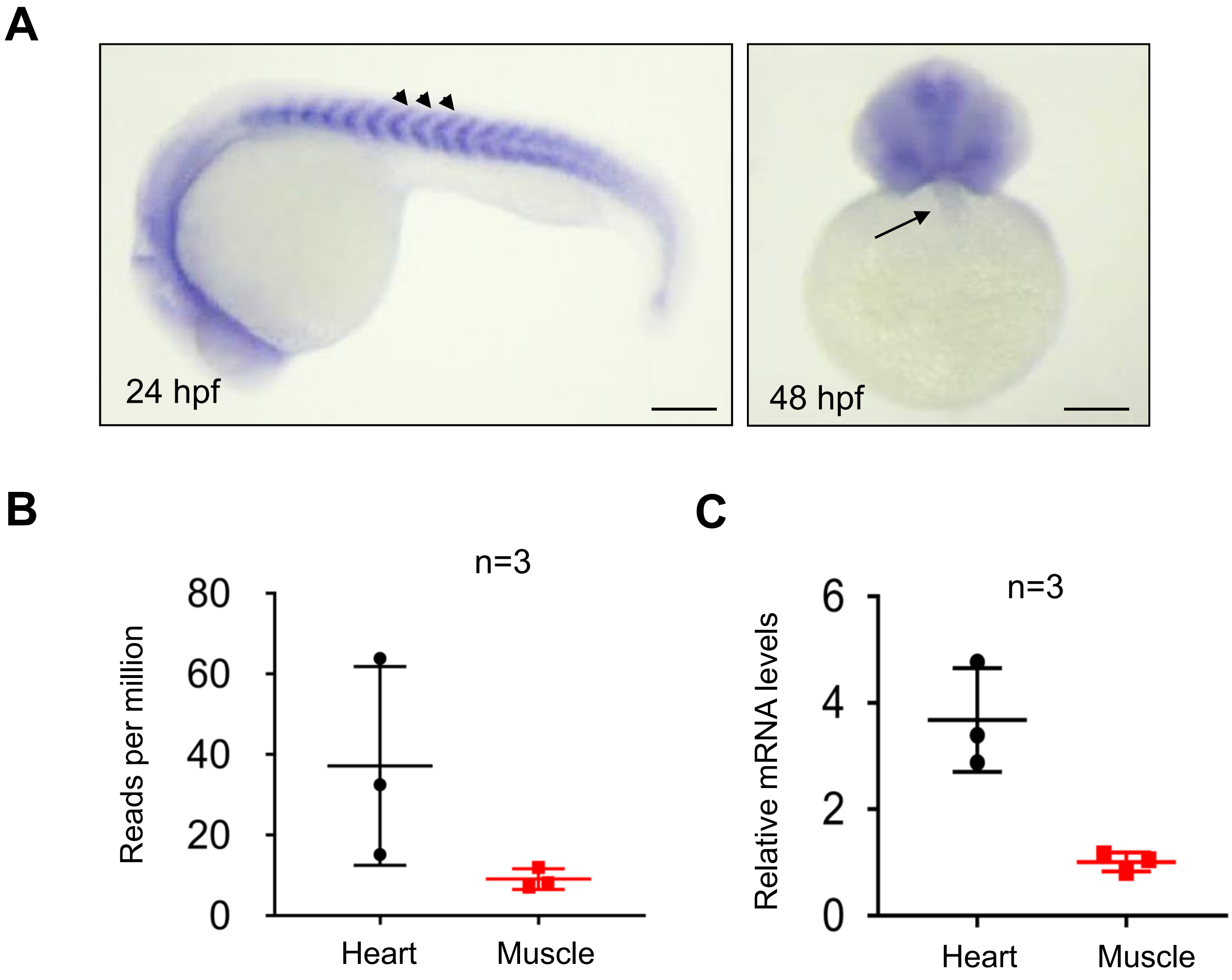

Fig. S2. Cardiac and somite expression of the *bag3* gene in zebrafish. (A) Whole-mount In Situ Hybridization showed *bag3* transcription is mainly expressed in the embryonic somites (arrowheads) and heart (arrow). hpf, hours post-fertilization. Scale bars: 200 µm. (B) RNA-seq analysis revealed that *bag3* is predominantly expressed in the heart tissue in 6-month-old adult zebrafish. (C) Quantitative RT-PCR results of relative *bag3* mRNA levels between the heart and muscle tissues in 6-month-old adult zebrafish.

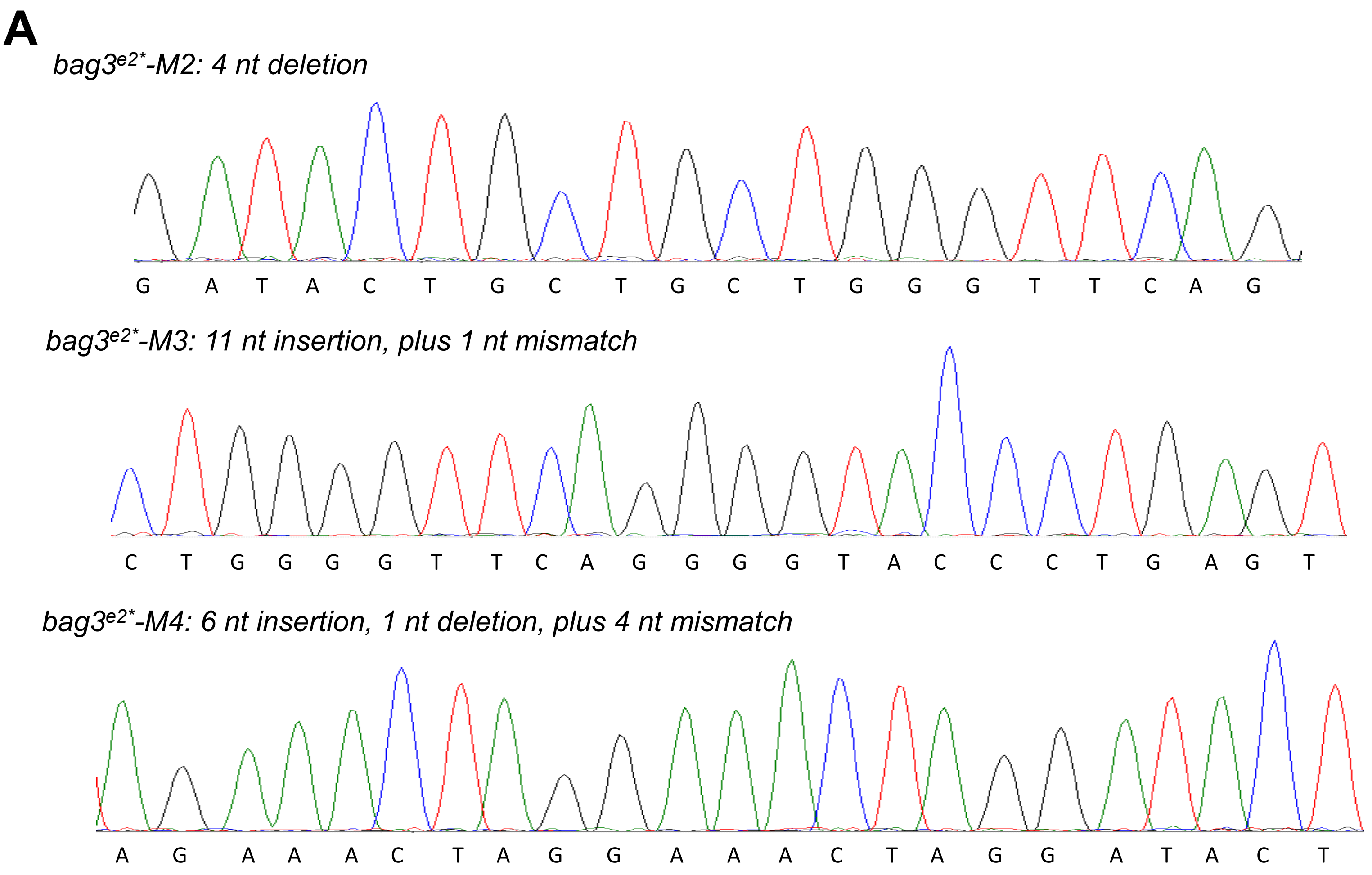

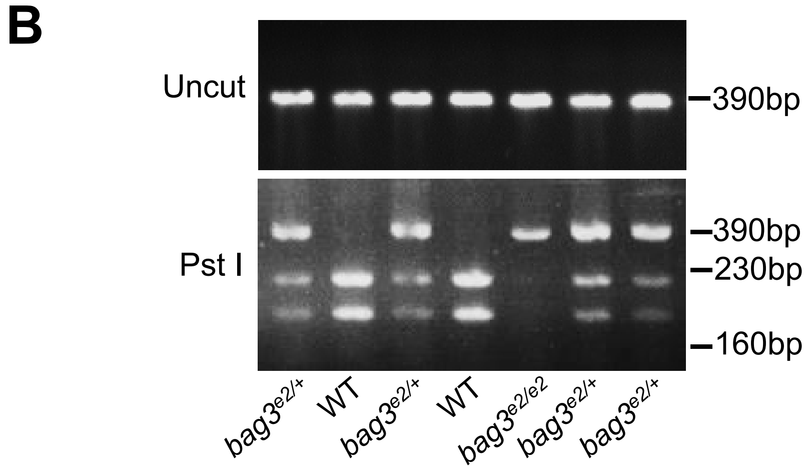

Fig. S3. Generation of different *bag3* mutant alleles. (A) Chromagraphs illustrate sequencing information of other three *bag3* mutant TALEN alleles depicted in Figure 1A . (B) Shown are examples of agarose gel images for the genotyping PCR results indicating the 10 nucleotides deletion allele of *bag3* heterozygous (*bag3*$^{e2/+}$), homozygous *(bag3*$^{e2/e2}$) mutants and WT control, respectively.

Disease Models & Mechanisms • Supplementary information

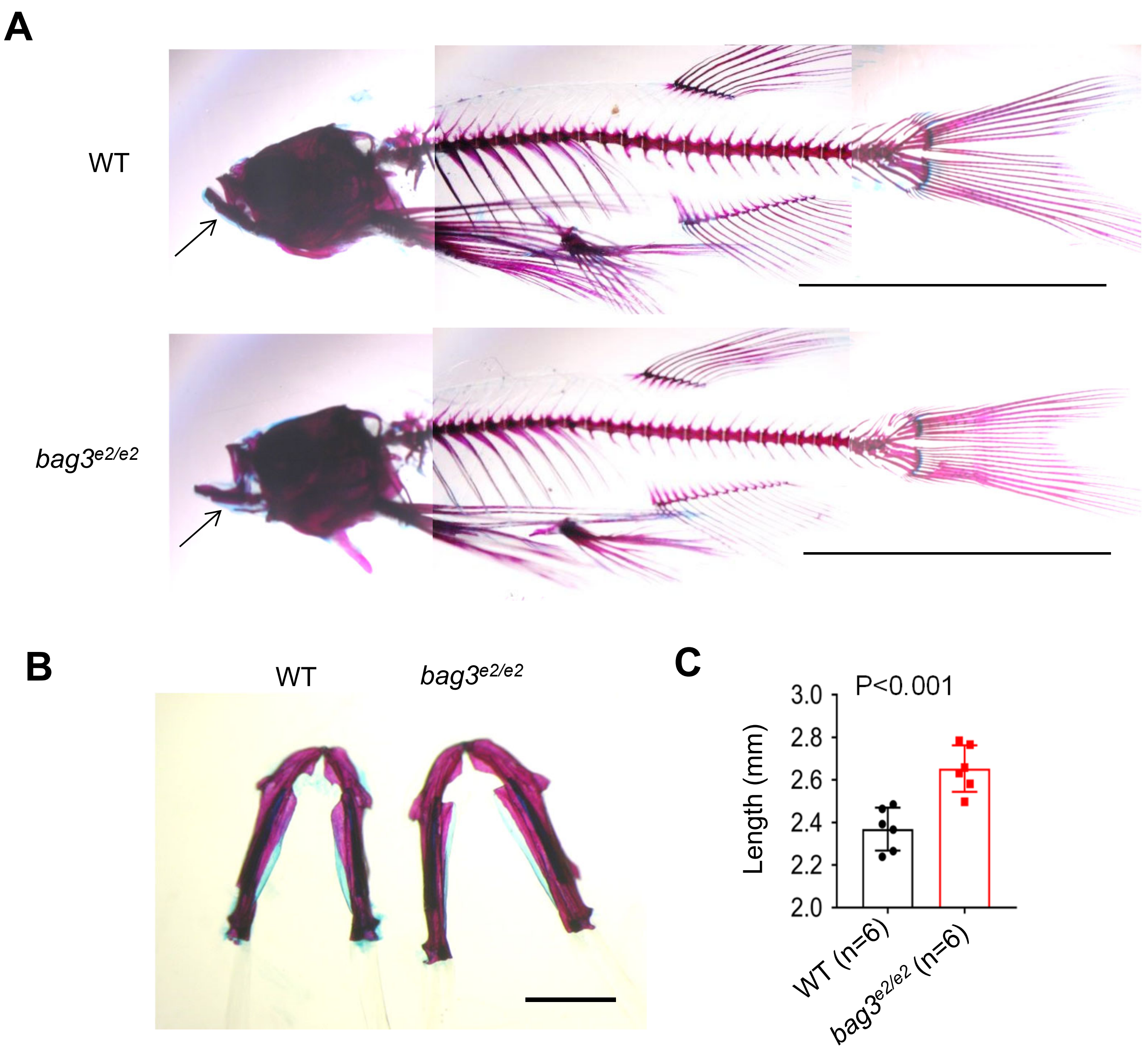

Fig. S4. The *bag3*$^{e2/e2}$ mutant exhibited elongated Meckel's cartilage phenotype. (A) Representative images of whole-mount staining for bones in the *bag3*$^{e2/e2}$ mutant fish and WT control at 3 months. The Meckel's cartilage in *bag3*$^{e2/e2}$ fish appears to be abnormal (arrows). Scale bars: 1 cm. (B) Representative images of dissected Meckel's cartilage from the *bag3*$^{e2/e2}$ mutant fish along with that from WT control at 3 months. Scale bar: 1 mm. (C) Quantification showed the length of Meckel's cartilage in the *bag3*$^{e2/e2}$ mutant is significantly longer than that in WT control. N=6, Student's *t* test.

Disease Models & Mechanisms • Supplementary information

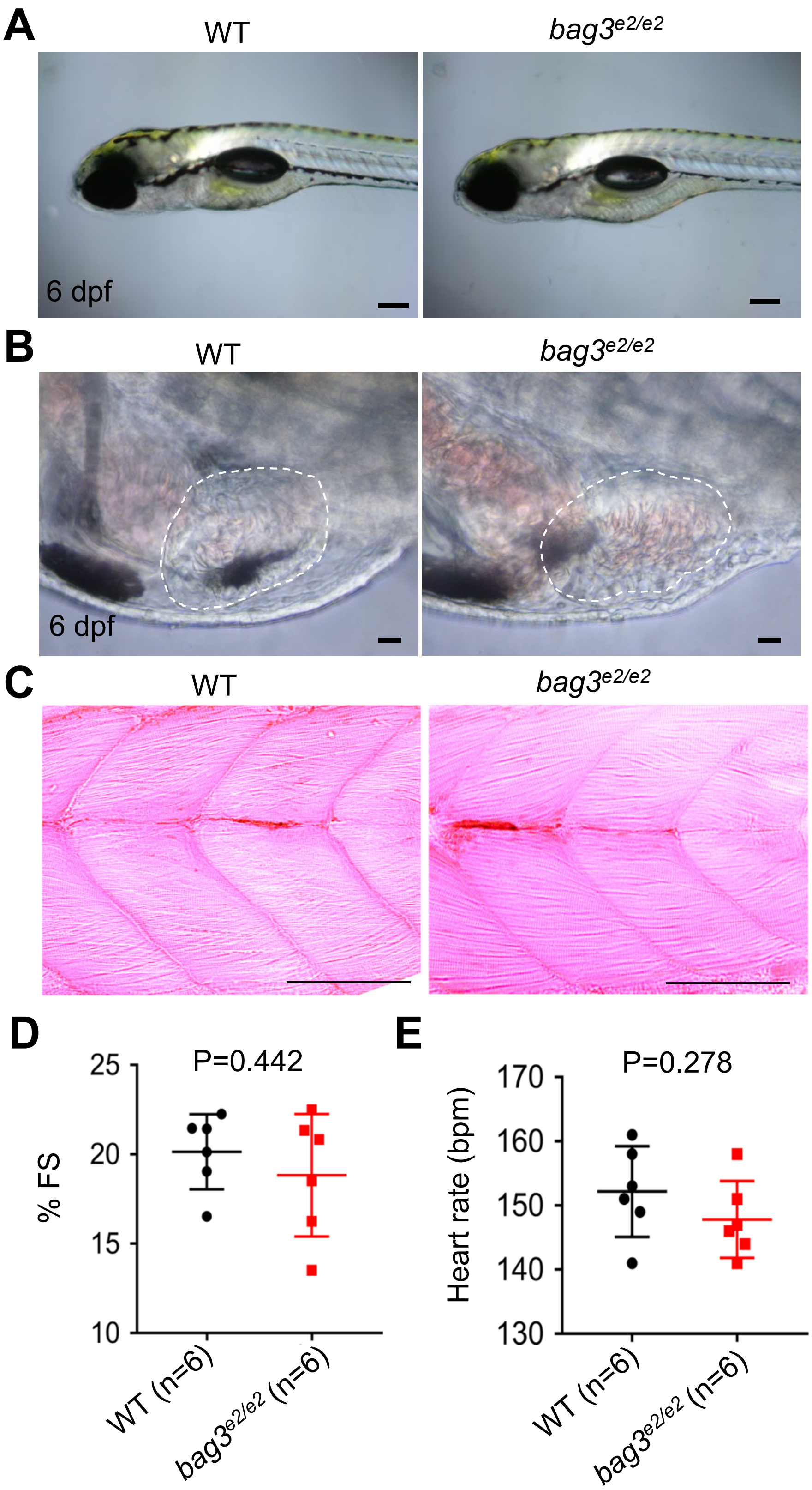

Fig. S5. No visible phenotypes were detected in the *bag3*$^{e2/e2}$ homozygous mutants r during early developmental stage (A) The embryonic shape of *bag3*$^{e2/e2}$ mutants appears to comparable to their WT siblings at 6 days post-fertilization (dpf). Scale bars, 200 µm. (B) The ventricular shape *bag3*$^{e2/e2}$ embryos appears to be comparable to their WT siblings at 6 dpf. Scale bars, 20 µm. (C), Representative images of H&E staining of somite in the *bag3*$^{e2/e2}$ mutants and WT controls at 6 dpf, Scale bars: 100 µm. (D-E) The fraction shortening (FS) (C) and heart rate (D) of *bag3*$^{e2/e2}$ embryos appear to be comparable to their WT siblings at 6 dpf. N=6, Student's *t* test.

Disease Models & Mechanisms • Supplementary information

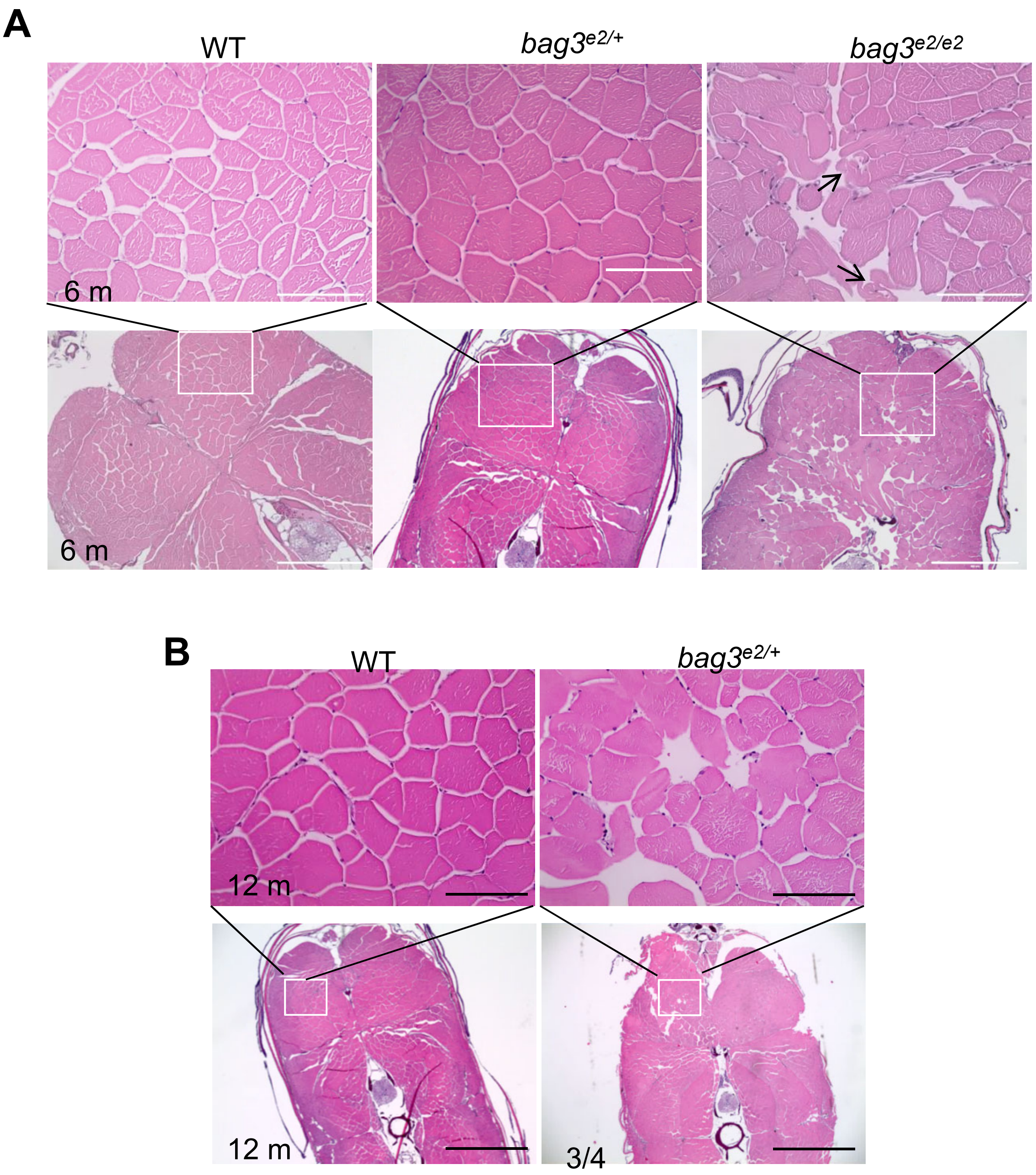

Fig. S6. Muscular abnormalities were detected in the *bag3* mutants at 6 and 12 months. (A) Representative H&E images of muscle from cross section showed signs of muscular disorganization phenotype (arrows) in the *bag3*$^{e2/e2}$ homozygous, but not *bag3*$^{e2/+}$ heterozygous mutants at 6 months. (B) Representative H&E images of muscle from cross section showed signs of loss of myofibril in the *bag3*$^{e2/+}$ heterozygous mutants at 12 months. Scale bars in upper panels: 100 μm, and lower panels: 1 mm.

Disease Models & Mechanisms • Supplementary information

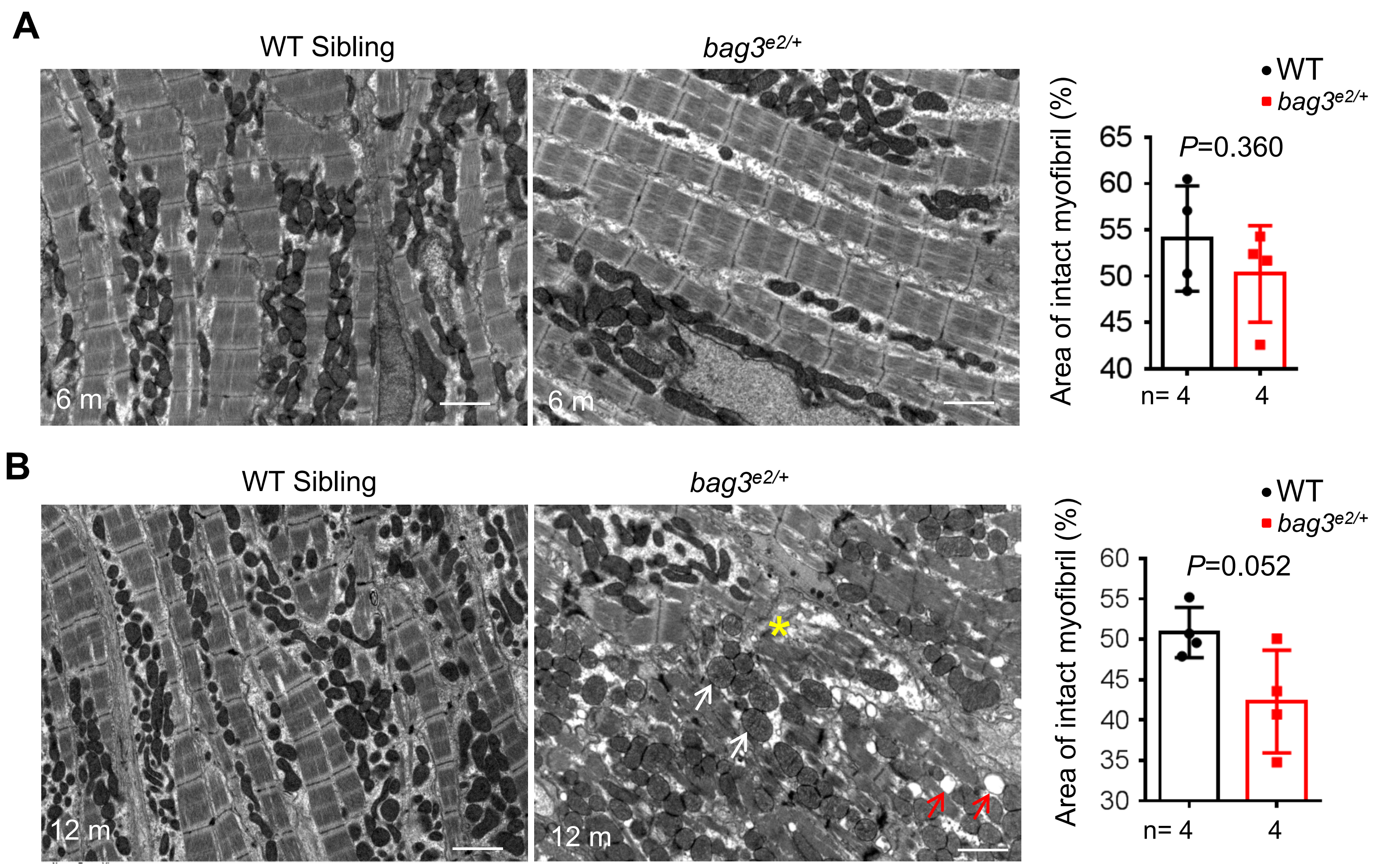

Fig. S7. The *bag3*$^{e2/+}$ heterozygous mutants exhibited cardiac abnormalities at 12 months, but not 6 months. (A-B) Confirmative TEM images of myofibril degeneration (yellow star) and mitochondrial swelling (white arrows) and/or vacuolization (red arrows) and quantification of intact myofibril area from the *bag3*$^{e2/+}$ heterozygous mutants compared with their corresponding WT siblings at 6 months (A) and 12 months (B). Scale bars: 2 μm. N=4, unpaired Student *t*-test.

Disease Models & Mechanisms • Supplementary information

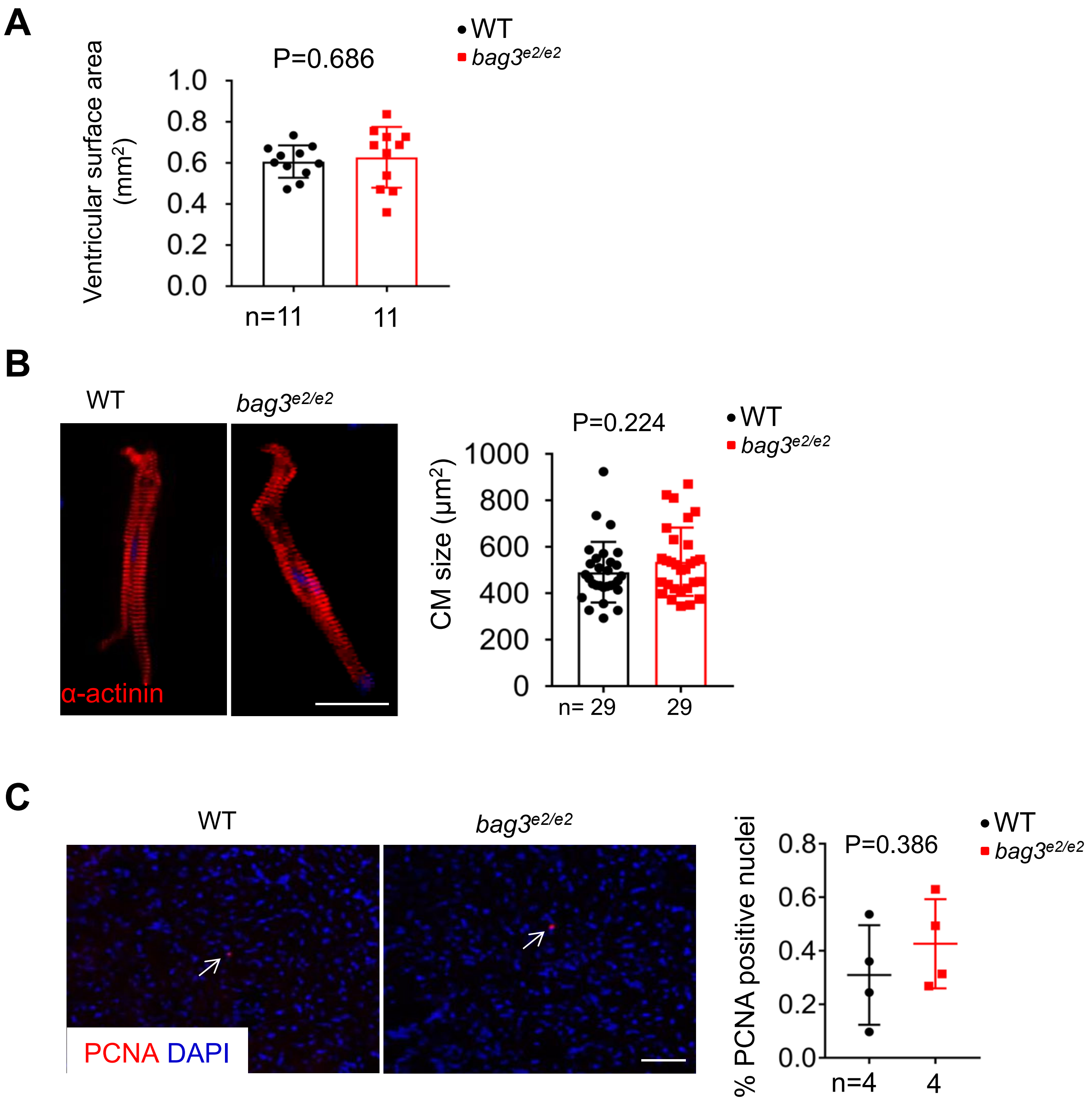

Fig. S8. Phenotypic characterization of the *bag3*$^{e2/e2}$ mutant. (A) Quantification of ventricular surface area (VSA) in the *bag3*$^{e2/e2}$ mutant compared to that in WT control at 6 months. n=11, Student *t* test. (B) Shown are representative images of anti-*α-actinin* antibody staining and cell size quantification of isolated single cardiomyocytes from WT and the *bag3*$^{e2/e2}$ mutant fish ventricle. Scale bar: 20 μm. n=29, Student's *t* test. (C) Representative images of PCNA staining counterstained with DAPI and quantification in the *bag3*$^{e2/e2}$ mutant hearts compared to those in WT control at 6 months. Scale bars: 20 μm. N=4, Student *t* test.

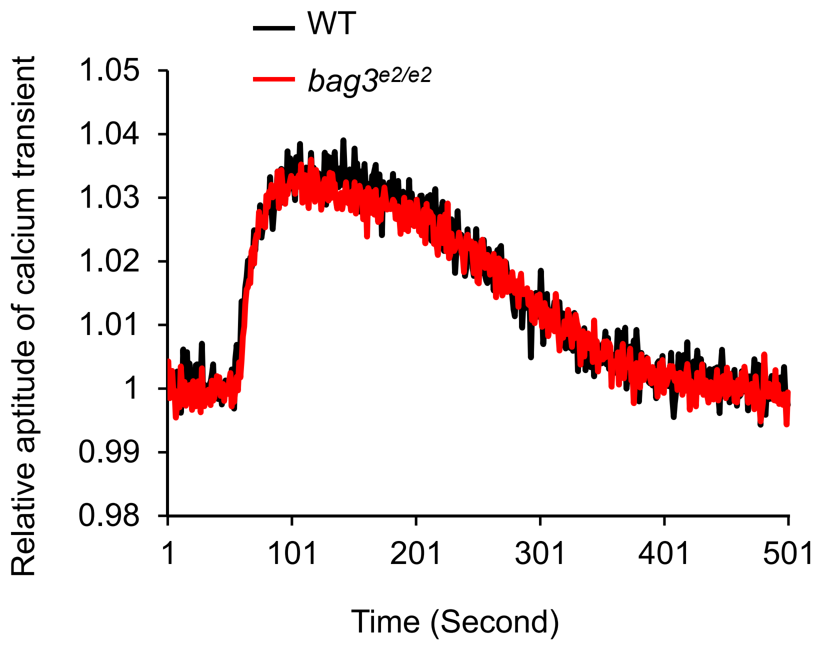

Fig. S9. Phenotypic characterization of the *bag3* mutant. No significant change of aptitude of calcium transient detected in single cardiomyocytes isolated from ventricle of the *bag3*$^{e2/e2}$ mutant compared to WT. WT: six cells were analyzed from four hearts. *bag3*$^{e2/e2}$: eleven cells were analyzed from three hearts.

Disease Models & Mechanisms • Supplementary information

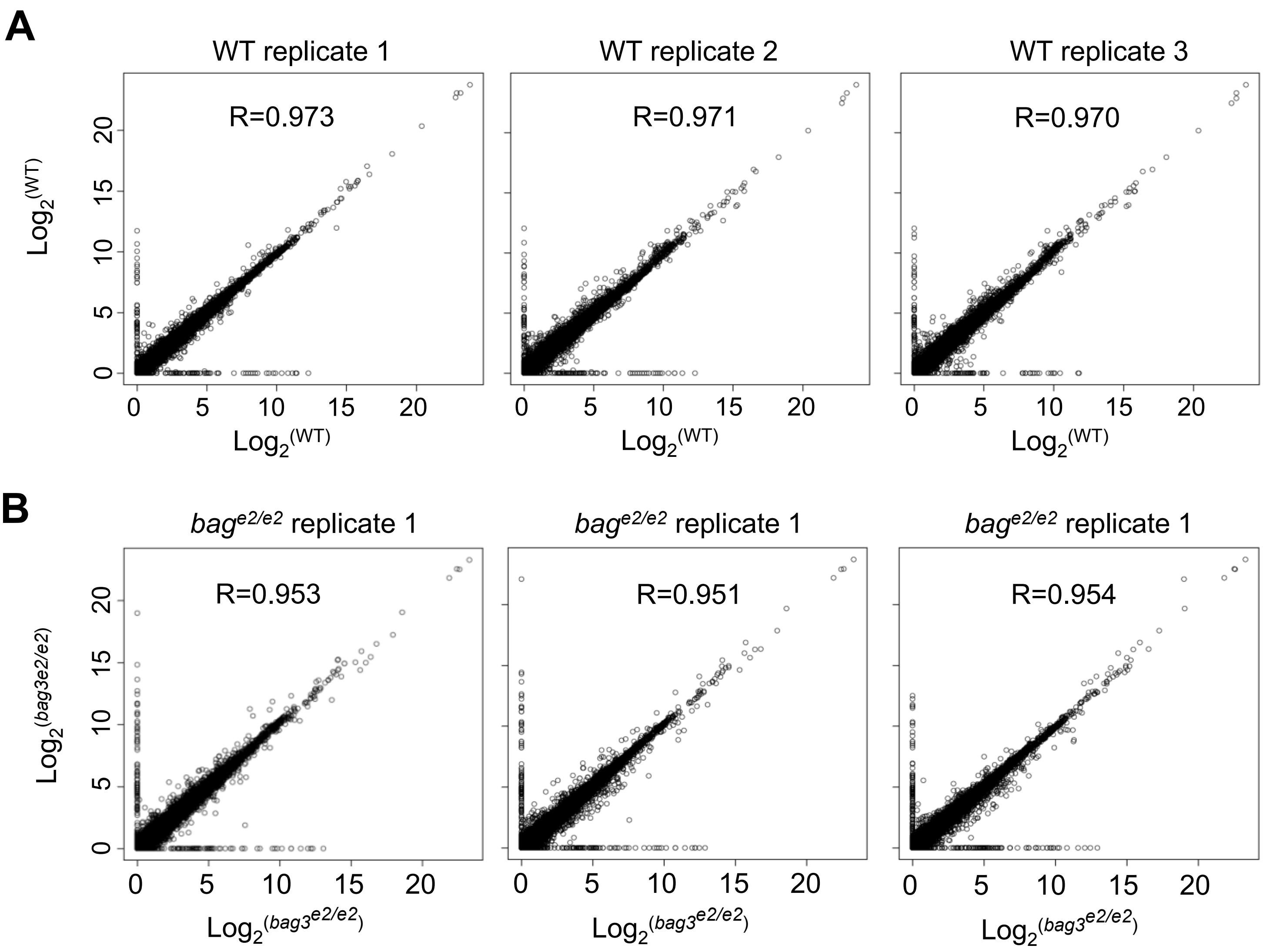

Fig. S10. Quality control of RNA sequencing datasets. (A-B) Scatter plots of Log2(FPKM+1) values between individual samples of three biological replicates for RNA sequencing of WT hearts (A) and *bag3*$^{e2/e2}$ hearts (B). The Pearson correlation coefficient (R^2) is shown in each comparison. FPKM: fragments per kilobase of transcript per million.

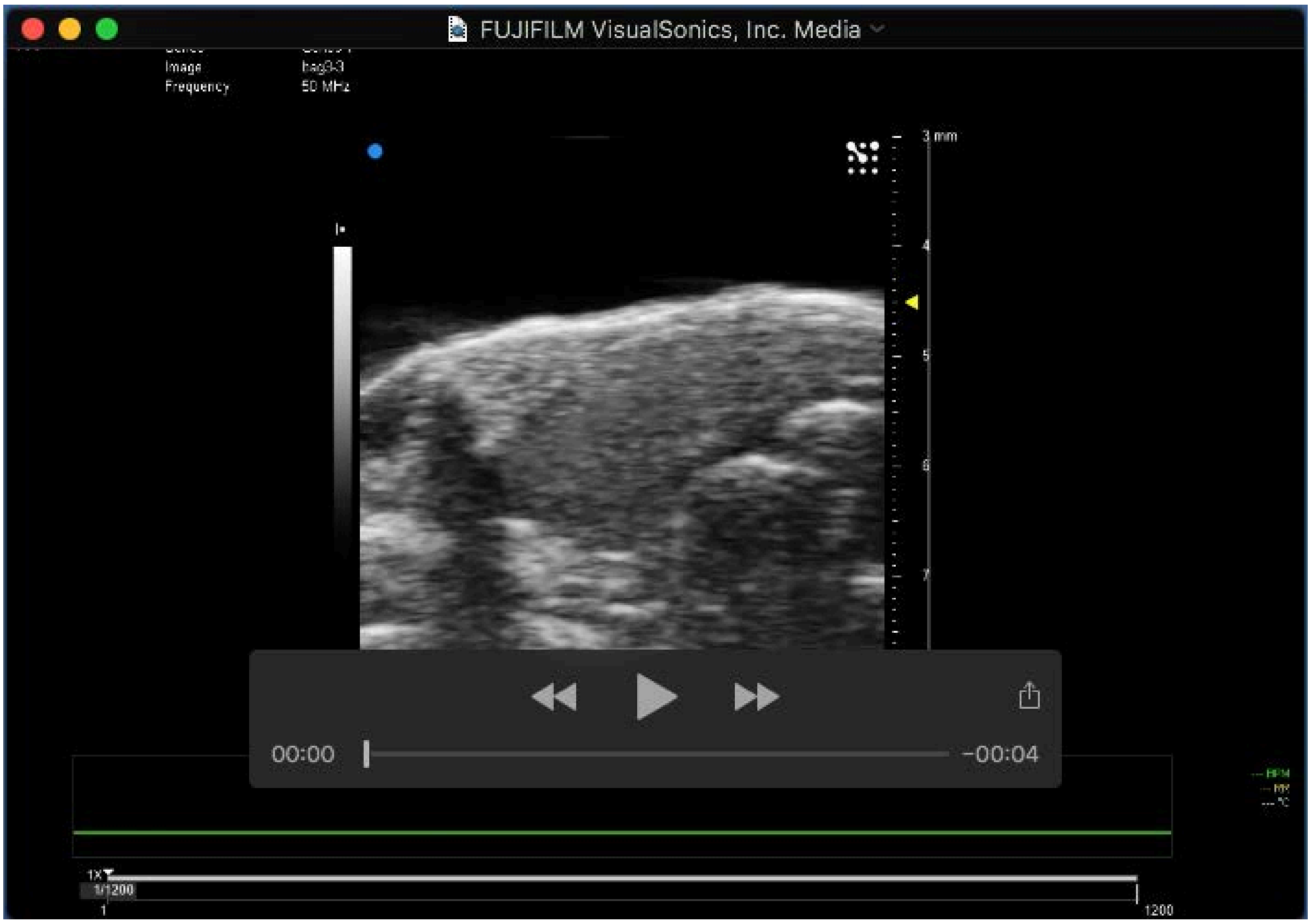

Movie 1_WT echocardiography

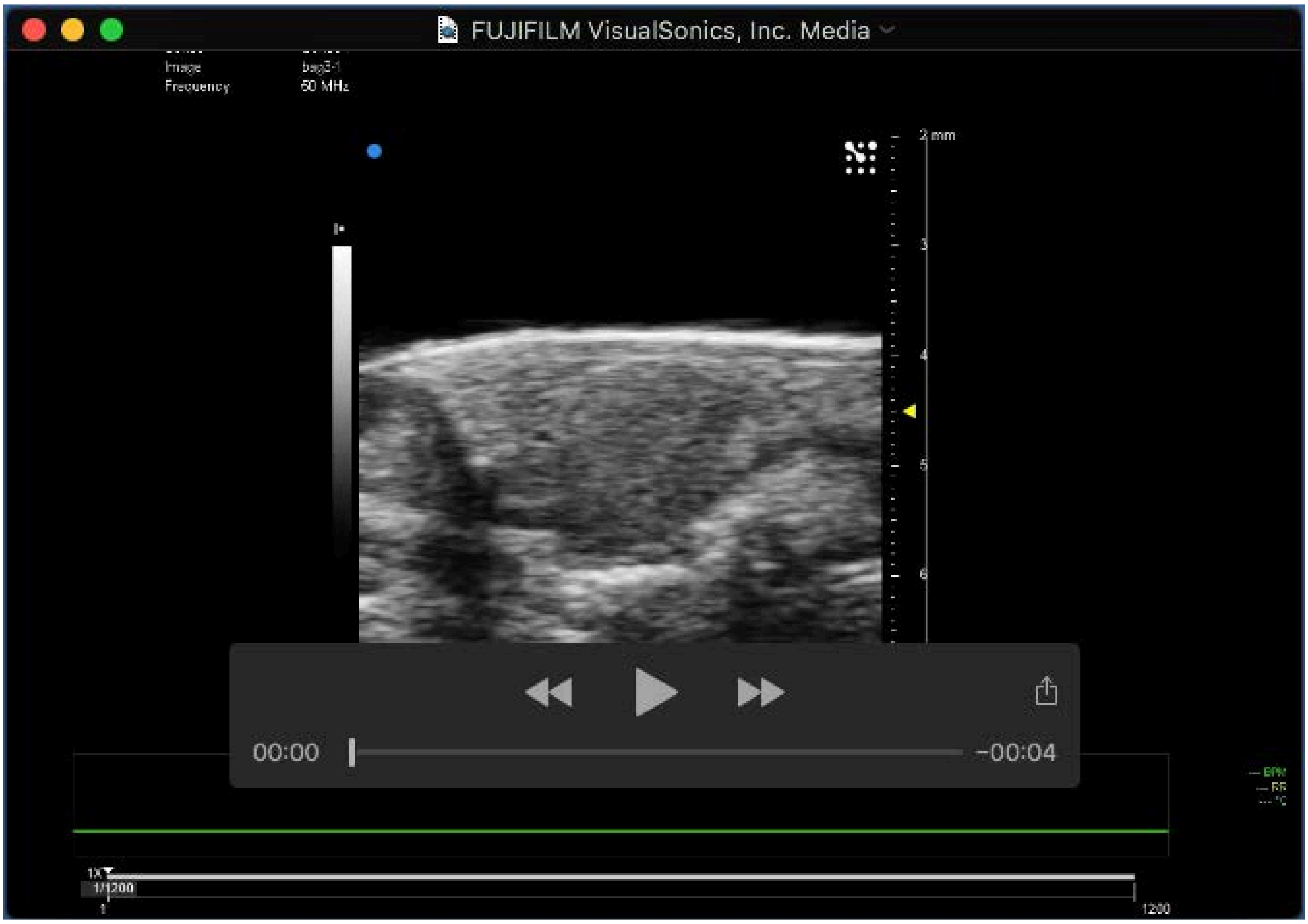

Movie 2_bag3 mutant echocardiography

Disease Models & Mechanisms • Supplementary information

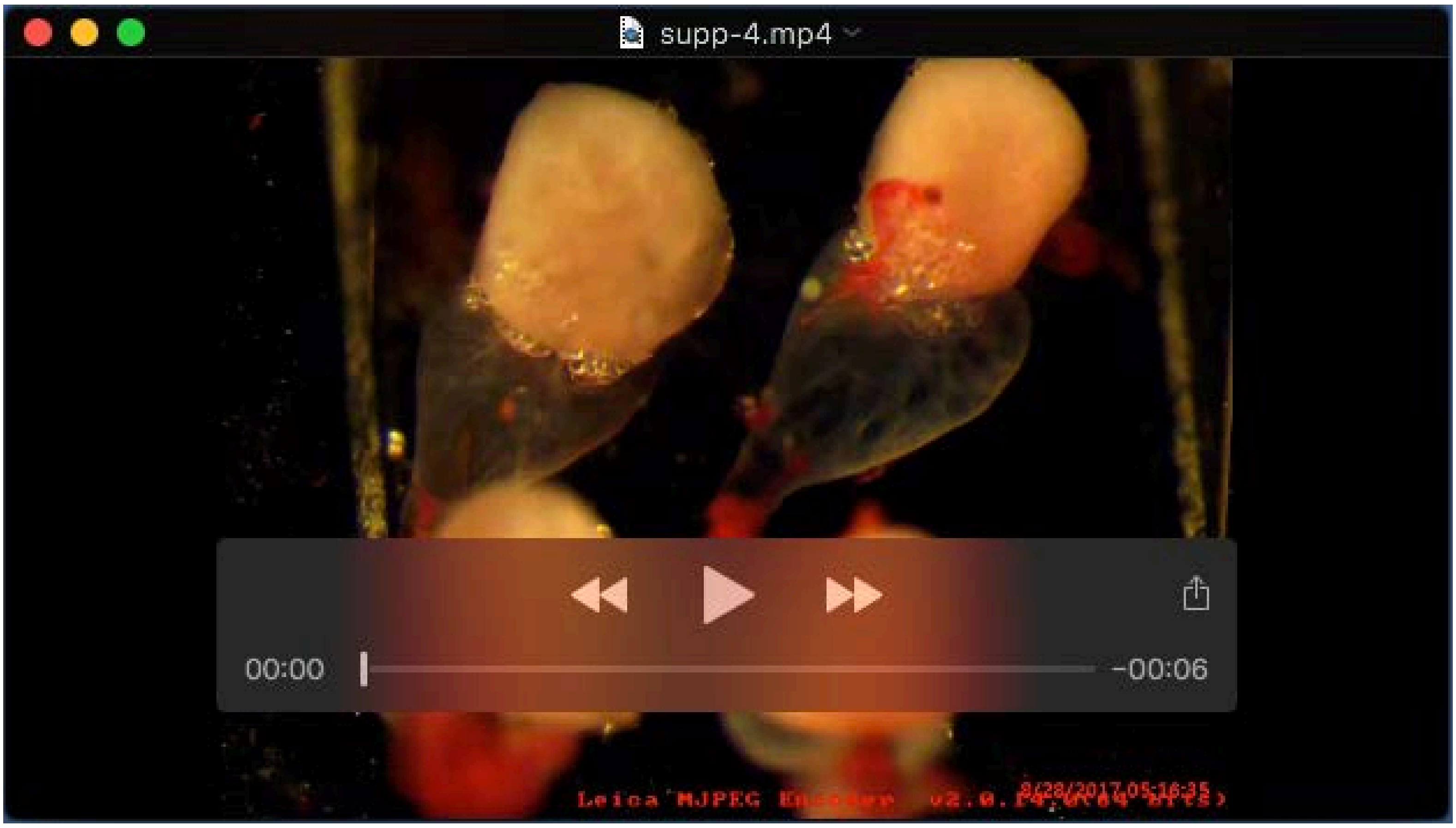

Movie 3_WT (right) and bag3 mutant (left) ex vivo

Disease Models & Mechanisms • Supplementary information
